# A Data-Adaptive Explainable Intrusion Detection Framework for IoMT Using Sampling Strategy Search and Ensemble Learning

**DOI:** 10.3390/s26217079

**Published:** 2026-11-09

**Authors:** Guzin Ulutas, Metehan Bulut, Beste Ustubioglu, Isilay Bozkurt, Mustafa Hakan Bozkurt, Dasara Shullani

**Affiliations:** 1 Department of Computer Engineering, Karadeniz Technical University, 61080 Trabzon, Türkiye; metehanbulut@ktu.edu.tr (M.B.); bustubioglu@ktu.edu.tr (B.U.); 2 Department of Software Development, Karadeniz Technical University, 61080 Trabzon, Türkiye; isilaybozkurt@ktu.edu.tr; 3 Department of Artificial Intelligence Engineering, Karadeniz Technical University, 61080 Trabzon, Türkiye; mhakanbozkurt@ktu.edu.tr; 4 Department of Information Engineering, University of Florence, 50139 Florence, Italy; dasara.shullani@unifi.it

**Keywords:** cyber security, ensemble learning, explainable artificial intelligence, intrusion detection system, Internet of Medical Things, IoMT security

## Abstract

In recent years, the widespread adoption of Internet of Medical Things (IoMT) systems in healthcare has provided significant advantages, particularly through rapid decision support and remote monitoring. At the same time, the growing number of heterogeneous and Internet-connected medical devices has expanded the cyberattack surface of healthcare infrastructures. Resource-constrained device characteristics, variable traffic patterns, and evolving attack types make fixed and conventional security mechanisms insufficient, especially for detecting previously unseen threats. This study proposes the Dynamic Data-Driven Pipeline (D3P), an adaptable and explainable intrusion detection framework for IoMT environments. Rather than relying on a fixed balancing strategy or a single predetermined model family, D3P automatically identifies an appropriate sampling strategy, selects the most successful learners from a candidate pool, performs model-specific feature selection, and constructs the final decision mechanism using a stacking-based meta-learner. The framework also incorporates explainability components based on SHapley Additive exPlanations (SHAP), Local Interpretable Model-agnostic Explanations (LIME), and BorutaSHAP to support both global and local interpretation. Experimental results on the test sets (mean ± standard deviation over five random seeds) show that D3P achieves 0.9701 ± 0.0015 accuracy, 0.9694 ± 0.0019 precision, 0.9701 ± 0.0015 sensitivity, and 0.9697 ± 0.0014 F1-score on WUSTL-EHMS-2020, while reaching 0.9978 ± 0.0004 accuracy, 0.9977 ± 0.0005 precision, 0.9978 ± 0.0005 sensitivity, and 0.9977 ± 0.0005 F1-score in the 6-class scenario of CICIoMT2024. These results indicate that D3P provides a balanced, reliable, and high-performance intrusion detection solution across IoMT datasets with different structural characteristics.

## 1. Introduction

In recent years, the use of the Internet of Medical Things (IoMT) has become increasingly widespread in healthcare, enabling continuous monitoring, remote diagnosis, data-driven treatment support, and faster clinical decision-making. As a result, IoMT has become an important component of contemporary healthcare systems [1,2]. Traditional healthcare services are being transformed into modern ecosystems supported by sensors, wearable devices, and smart medical equipment, with IoMT networks serving as the backbone of this transition [1,2]. However, the increase in the number of interconnected and Internet-connected medical devices has also expanded the attack surface of healthcare infrastructures. In addition, IoMT devices are typically resource-constrained in terms of power, memory, and computational capacity, which limits the range of security mechanisms that can be deployed. These constraints make IoMT environments particularly vulnerable to cyber threats [3,4,5].

Because emerging cyber threats target not only data assets but also the continuity of medical services, cybersecurity in IoMT networks is a more critical concern than in many other IoT domains. Threats such as denial-of-service attacks, ransomware, and malware can interrupt healthcare communication processes and compromise the integrity of medical information [3,4,6,7]. Considering the heterogeneous and resource-limited nature of IoMT environments, relying solely on conventional security controls such as authentication, encryption, and access control is often insufficient, particularly against evolving and previously unseen zero-day attacks [4,5]. For these reasons, anomaly-based intrusion detection systems (IDSs), which monitor traffic behavior and detect suspicious activities in real time, have received increasing attention in recent years, especially for zero-day attack detection [3,4,6].

A key advantage of anomaly-based IDSs over rule-based approaches is their ability to identify and classify attack patterns that were not explicitly defined beforehand. In the IoMT literature, anomaly detection models are implemented using both classical machine learning and deep learning techniques. Recent studies report hybrid and ensemble solutions that include recurrent architectures (e.g., SRU, LSTM, and BiLSTM), as well as Transformer-based and boosting-based approaches [3,4,7,8,9]. These methods demonstrate the ability to learn complex traffic patterns and temporal dependencies from medical network data across widely used benchmark datasets [3,4,7,9].

Alongside improvements in detection performance, explainable artificial intelligence (XAI) has become increasingly important in this field. In healthcare-related cyber environments, understanding why a model flags an event as malicious is essential due to strict transparency and interpretability requirements. Therefore, many recent IoMT intrusion detection studies integrate SHAP- and LIME-based explanation mechanisms to provide post hoc interpretation and improve trust in model decisions [5,6,7,8,9,10,11].

Despite these developments, several limitations remain:

- Class imbalance persists in both benchmark datasets and real-world network traffic. Most studies address this issue through fixed or limited balancing strategies instead of systematically searching for the most suitable sampling configuration for a specific dataset [3,6,7,11]. However, the effectiveness of a balancing strategy can vary substantially across datasets and attack scenarios, especially in highly skewed IoMT data.
- Many existing methods rely on relatively stable model designs, even when ensemble or hybrid frameworks are used [4,8,9]. In many cases, learning strategies are not adaptively determined according to traffic characteristics, and the model setup remains fixed without sufficiently reflecting network-specific conditions. Such methods often assume a fixed data representation and may face architectural incompatibilities when applied to different environments.
- Although feature selection and explainability are widely studied, feature optimization is often handled at a global level; model-specific feature selection within multi-learner frameworks remains comparatively underexplored [5,6,10,11].

Motivated by these gaps, this study (Dynamic Data-Driven Pipeline, D3P) proposes an adaptable IDS framework for IoMT environments that integrates data balancing, model selection, feature optimization, and final ensemble construction within a unified architecture. Instead of depending on a predefined balancing method or a fixed classifier family, the framework identifies an effective over-sampling/under-sampling configuration for the target dataset. It then selects suitable base learners from a candidate pool, combines them through a meta-learning stage, and dynamically determines the meta-learner. In addition, a BorutaSHAP-based feature selection module identifies learner-specific sub-feature spaces. The framework also includes optional global and local explanation mechanisms based on SHAP and LIME to support interpretability.

The main contributions of D3P can be summarized as follows. First, an adaptive sampling-search mechanism is introduced to determine effective balancing configurations for highly imbalanced IoMT datasets. Second, the framework dynamically selects both base learners and meta-learners instead of relying on a single fixed model family. Third, BorutaSHAP-based feature selection is integrated to identify highly informative input features. Finally, an optional XAI layer provides both global and local explanations for transparent interpretation of detection outcomes. The effectiveness of the proposed framework is also supported by the experimental findings obtained on two different IoMT datasets. On the test set of WUSTL-EHMS-2020, the proposed D3P architecture achieves 97.01 ± 0.15% accuracy, 96.94 ± 0.19% precision, 97.01 ± 0.15% recall, and 96.97 ± 0.14% F1-score (mean ± standard deviation over five random seeds), outperforming the strongest standalone base learners on the validation subset. On the 6-class CICIoMT2024 dataset, D3P further reaches 99.78 ± 0.04% accuracy, 99.77 ± 0.05% precision, 99.78 ± 0.05% recall, and 99.77 ± 0.05% F1-score, demonstrating that the proposed adaptive pipeline can produce highly competitive and balanced results across datasets with different structural properties. These findings indicate that the joint optimization of sampling, learner selection, feature selection, and stacking-based fusion provides not only methodological flexibility but also measurable practical gains for IoMT intrusion detection.

The remainder of this paper is organized as follows. Section 2 reviews recent studies on IoMT intrusion detection, covering both traditional rule-based methods and artificial intelligence-based anomaly detection approaches. Section 3 presents the proposed adaptive and explainable framework in detail, including data preprocessing, sampling-strategy selection, base learner selection, feature optimization, and final stacking-based ensemble construction. Section 4 reports the experimental setup and results on the WUSTL-EHMS-2020 and CICIoMT2024 datasets, together with intermediate analyses and comparative evaluations. Section 5 discusses the results in comparison with previous studies, and finally, Section 6 summarizes the main findings and outlines possible future research directions.

## 2. Related Work

In the literature on intrusion detection, existing studies can be grouped into two main categories: rule-based approaches and artificial intelligence-based anomaly detection systems. In this section, the strengths and limitations of both categories are discussed, drawing on recent studies from each line of research.

### 2.1. Traditional and Rule-Based Detection Methods

In the literature, signature-based intrusion detection methods are among the most widely recognized attack detection approaches [12,13]. The most important advantage of these rule-based methods is their relatively fast and effective operating architecture, since they search incoming traffic for predefined traces. These systems can produce high accuracy for known attacks while maintaining relatively low false alarm rates. For these reasons, signature/rule-based detection is still widely used today in open-source solutions such as Snort and Suricata, as well as in commercial products such as Palo Alto Networks Threat Prevention. However, the most significant disadvantage of rule-based approaches is that, by nature, they query network traffic against an existing signature database. Therefore, these solutions may become ineffective in the presence of zero-day, variant, or previously unseen threats [12,13].

This limitation is more pronounced in IoMT networks, because medical devices in these environments are heterogeneous and involve different protocols, vendor-specific communication patterns, and continuously changing traffic behavior. The dynamic nature of these networks makes rule-based approaches harder to maintain and deploy [14]. In the IoMT ecosystem, the continuous emergence of new threats, the resource-constrained nature of devices, and the operational burden of frequent database updates and deep packet inspection further reduce the practicality of rule-based methods. Therefore, in the current literature, signature-based methods are generally considered a baseline or complementary defense layer, while the main trend shifts toward anomaly-based and AI-supported IDS approaches discussed in Section 2.2.

### 2.2. Artificial Intelligence-Based Anomaly Detection Methods

In IoMT networks, heterogeneity among devices, data imbalance (i.e., the relatively limited amount of attack samples), and the continuously evolving nature of attack types have pushed intrusion detection research away from conventional approaches toward artificial intelligence-based strategies. Accordingly, the literature shows that AI-driven anomaly detection methods have become increasingly popular among researchers. Broadly, existing studies can be grouped into two categories: (i) classical machine learning approaches that make decisions based on hand-crafted or selected features, and (ii) deep learning approaches that can automatically learn representations from raw or transformed data. Classical machine learning methods usually offer lower computational cost and can provide strong performance when feature selection is effective. Although deep learning methods are more computationally demanding, their ability to model non-linear and temporal patterns often yields highly competitive detection performance. In this section, representative studies published in the last three years are briefly summarized for both classical ML-based IDSs and recent DL-based IDS approaches.

#### 2.2.1. Classical Machine Learning (ML) Approaches

Among methods proposed for attack detection in IoMT environments, classical machine learning approaches stand out due to their decision efficiency and implementation simplicity. Most studies in this domain perform classification using selected network and/or biometric features and aim to achieve high accuracy with relatively low computational overhead. Representative works are summarized below.

Balhareth and Ilyas [15] combined tree-based machine learning and filter-based feature selection to improve efficiency and accuracy in real-time intrusion detection. Their study evaluated classical classifiers such as Decision Tree, Random Forest, XGBoost, and CatBoost, while feature selection jointly considered Mutual Information and XGBoost-based feature importance rankings. Experimental results on CICIDS2017 reported 98.79% accuracy and a 0.007 false alarm rate, indicating that high performance can be achieved with fewer features.

Oragwu et al. [16] focused not only on attack detection but also on identifying lifecycle stages at which IoMT devices become more vulnerable. Using CICIoMT2024, the proposed method built a three-stage evaluation framework: a classifier for device lifecycle status, a classifier for device-specific attack types, and an analysis layer visualizing vulnerabilities across lifecycle stages. Random Forest achieved 0.8915 accuracy in lifecycle classification and 0.9789 accuracy in device/attack-context classification.

Rehman et al. [17] investigated the relationships among feature subsets for ML-based IDS in IoMT. Using CICIoMT2024 and IoMT-TrafficData datasets, filter-based feature selection methods (Fisher Score, Mutual Information, Information Gain) were evaluated with XGBoost, KNN, Decision Tree, and Random Forest. Performance comparisons across binary and multi-class scenarios showed that even 3–4 features can yield strong results for binary classification, while 7–8 features are often sufficient for multi-class settings. The authors reported that the Information Gain + XGBoost + 15-feature combination produced robust performance in both binary and multi-class scenarios.

Ibrahim and Al-Wadi [18] proposed an ensemble learning strategy using Random Forest, XGBoost, AdaBoost, MLP, Logistic Regression, and KNN. The study evaluated bagging, boosting, stacking, and weighted voting; the best performance was obtained with a weighted voting ensemble composed of LR + ADA + XGB. The final model achieved 0.9960 accuracy in 12.42 s.

Domènech et al. [19] compared CICIoT2023 and CICIoMT2024 datasets and examined how well ML-based IDS models generalize when trained on one dataset and tested on another. Experiments involved Decision Tree, Random Forest, XGBoost, and a feedforward neural network, with explicit analysis of dataset shift. Results showed that cross-dataset evaluation can lead to F1-score drops of up to 66.87%.

Pinar et al. [20] proposed a new feature selection framework based on RSFS + CFS to strengthen threat detection in IoMT cybersecurity. Using WUSTL-EHMS-2020, TON-IoT, ICU-Dataset, and ECU-IoHT, they performed comprehensive experiments with Random Forest, KNN, SVM, XGBoost, and DNN. The approach achieved 99.82% on TON-IoT, 99.99% on ICU-Dataset, 96.37% on WUSTL-EHMS-2020, and 99.99% on ECU-IoHT.

Hafid et al. [21] emphasized that interpretability and cost-awareness should also be considered in IoMT intrusion detection. Their study compared a carefully tuned XGBoost model with a regularized Logistic Regression baseline and then combined them through a max-voting late-fusion mechanism. SHAP was also used to analyze key features driving model decisions. The proposed late-fusion structure reached precision = 0.98.

Kouassi et al. [22] argued that using too many features introduces unnecessary computational burden in IoT/IoMT intrusion detection and therefore used a Top-K feature selection strategy. On CICIoMT2024, XGBoost, LightGBM, and Random Forest were evaluated under Top-10, Top-15, and full-feature configurations. Feature importances from three ensemble learners were aggregated and combined through voting to select the most discriminative variables. The study reported that Random Forest with Top-10 features provided the best trade-off, achieving 91.7% accuracy and 93% F1-score.

#### 2.2.2. Deep Learning (DL) Approaches

Deep learning-based approaches for IoMT intrusion detection have gained increasing importance in recent years. These studies typically model spatial and temporal dependencies using deep and hybrid architectures, aiming for stronger detection performance on multi-class IoMT data. Representative works are summarized below.

Ravi et al. [23] proposed a CNN-LSTM-based IDS that combines patient biometric data with network-derived features. Their architecture employed a global attention mechanism to emphasize spatio-temporal patterns and used cost-sensitive learning to mitigate class imbalance. On WUSTL-EHMS-2020, they reported 95% accuracy with network features, 89% with biometric features, and 99% with combined features. Using 10-fold cross-validation, the study incorporated not only network traffic but also patient biometric information into IDS decision-making.

Faruqui et al. [24] introduced SafetyMed, a CNN-LSTM hybrid IDS that leverages both sequential traffic data and image-based representations. The framework also includes an edge-server decision mechanism capable of producing a response when an attack is detected. On CICIDS2017, the reported results were 97.63% accuracy, 98.47% precision, 97% recall, and 97.73% F1-score.

Naeem et al. [25] proposed an architecture combining data augmentation, feature selection, and a stacking-based deep ensemble. Transformer-based networks, self-attention DCNN, and LSTM were trained as base learners, and their outputs were fused with a meta-learner. A BAT algorithm was used for data augmentation. Evaluation on WUSTL-EHMS-2020 and CICIoMT2024 reported 100% and 99% accuracy, respectively. This study is an important example of ensemble fusion and multi-model learning in DL-based IoMT IDSs.

Rbah et al. [26] proposed a hybrid GNN-BiLSTM model. The graph-based component captures inter-device relationships and network topology, while the Bi-LSTM component learns temporal patterns in network flows. On the IoT-Healthcare security dataset, the model achieved 99.98% accuracy, 99.98% precision, and 100% recall.

In [27], an LSTM-based model was used to detect multi-class attacks in CICIoMT2024. Experimental results showed that the proposed method achieved 98% accuracy for 19 classes.

Zukaib et al. [28] proposed Meta-IDS, a meta-learning-based architecture. The system first performs signature-based detection using weak learners and a meta-learner, then supports anomaly-based detection through mean-shift clustering and biased classifiers. For hyperparameter optimization, Bat Algorithm and a tree-structured Parzen estimator-based approach were used. Tested on WUSTL-EHMS-2020, IoTID20, and WUSTL-IIOT-2021, the model reported 99.57%, 99.93%, and 99.99% accuracy for signature-based detection, and 99.47%, 99.98%, and 99.99% for anomaly-based detection.

Benahmed et al. [29] introduced HBiLD-IDS, which uses a BiLSTM-DNN hybrid model for real-time intrusion detection in IoMT networks. The method combines BiLSTM’s capability to learn bidirectional temporal dependencies with DNN’s hierarchical feature extraction power. On CICIoMT2024, the model reported 98.81% classification accuracy across 19 attack types.

Sharma and Shambharkar [30] proposed MA-DeepCRNN, where CNN captures spatial patterns and RNN/Bi-LSTM components model temporal dependencies. A multi-attention mechanism helps focus on the most critical traffic patterns. The study also incorporated interpretability methods and analyzed results accordingly. On CICIoMT2024, the architecture achieved 99.49% for binary classification, 99.12% for 6-class classification, and 98.56% for 19-class classification.

To position the proposed D3P architecture more clearly in the recent IoMT-IDS landscape, a methodological comparison is provided in Table 1. Rather than comparing only predictive scores, this table evaluates whether each study satisfies five practical criteria that directly affect deployment value: data adaptivity, explainability, imbalance handling, cross-dataset validation, and reproducibility.

In this comparison, data adaptivity refers to whether the approach can dynamically adjust key pipeline decisions (e.g., selecting feature-selection or balancing strategies). Explainability indicates the use of XAI tools such as SHAP/LIME. Imbalance handling denotes explicit treatment of skewed class distributions. Cross-dataset validation captures whether the method is tested on more than one dataset. Reproducibility reflects the availability of sufficient implementation and parameter details. As shown in Table 1, D3P is the only method in this set that jointly satisfies all five criteria, indicating a balanced contribution not only in performance but also in methodological completeness and practical usability.

## 3. Proposed Framework

**Figure 1:**
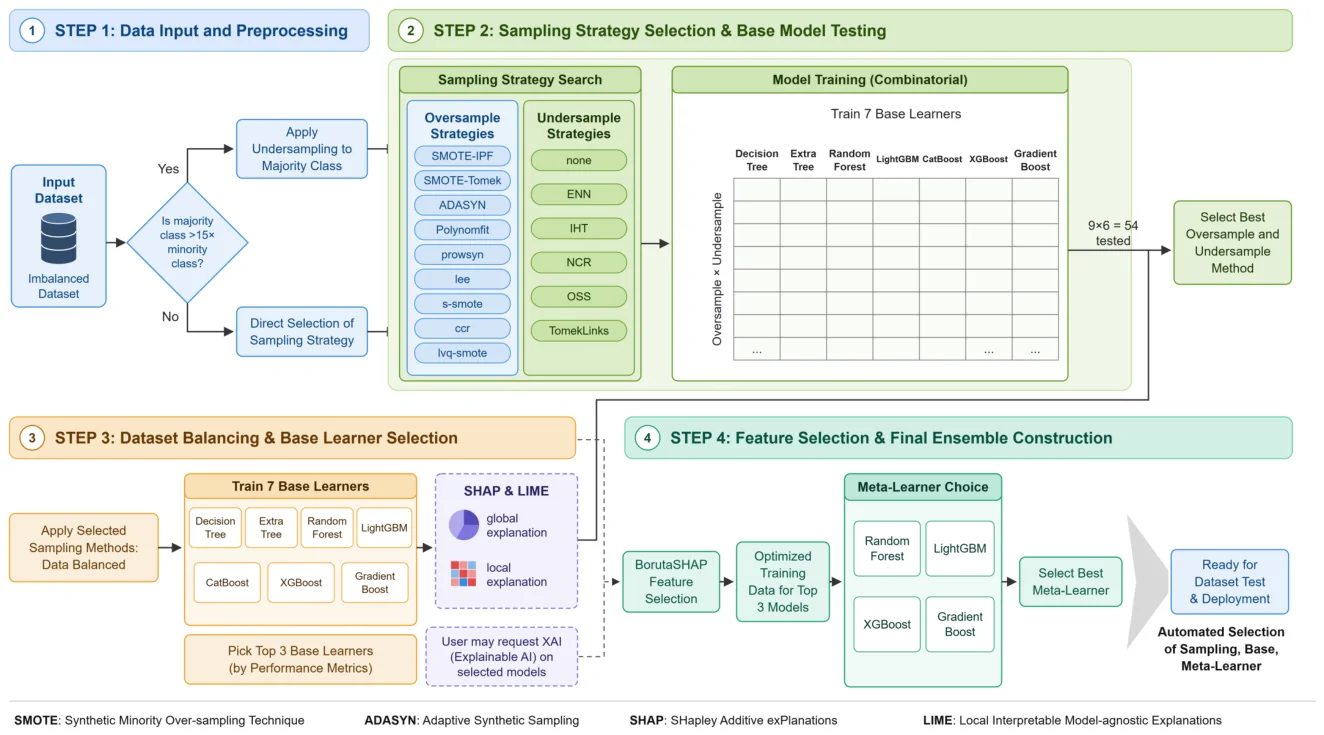
Overall workflow of the proposed dynamic and adaptive intrusion detection framework.

The proposed D3P framework aims to detect attacks in imbalanced and structurally variable IoMT networks. Its overall workflow is presented in Figure 1 and consists of four steps: data input and preprocessing, sampling-strategy selection, dataset balancing and evaluation of base learners (selection of the top three), and feature selection with construction of the final ensemble architecture. Unlike conventional approaches that feed the input data into a fixed processing pipeline, D3P constructs these steps for each dataset, so that it can be applied to any network for which a labeled dataset is available.

The framework addresses class imbalance, high-dimensional or noisy features, performance variability caused by manually selected models, and limited explainability of model decisions. It first analyzes the imbalance ratio of the dataset and applies preliminary reduction when needed (Section 3.1). Different oversampling and undersampling strategies are then evaluated combinatorially, and the most suitable balancing pair is selected for the target dataset. Next, the base learners are compared on the balanced data, the three best-performing learners are selected, and their behavior can optionally be examined with SHAP, LIME, or BorutaSHAP explanations. Learner-specific feature subsets are then chosen by comparing RFE, L1/Lasso, and BorutaSHAP, and the selected base learners are finally combined in a stacking-based ensemble whose meta-learner is selected from four candidates. Section 3.1, Section 3.2, Section 3.3 and Section 3.4 describe these four steps in detail.

### 3.1. Step 1: Data Input and Preprocessing

In the first stage of the proposed method, the dataset received by the system is subjected to a preliminary evaluation in terms of class distribution. An IDS intended to operate in any environment should be able to accept a dataset generated from the current network regardless of whether its class-level distribution is balanced. Let the input dataset be denoted by *D*. For a problem with *K* classes, this dataset can be represented as the union of *K* disjoint class subsets $D_{1},\ldots ,D_{K}$, as given in Equation (1). In the WUSTL-EHMS-2020 setting, K=2 (benign and attack), whereas in CICIoMT2024, K=6, with the individual attacks grouped into five attack categories and a benign class, in line with the common practice of organizing intrusions into attack groups [49].

(1) $$D=\overset{K}{\underset{c=1}{⋃}}D_{c},\;D_{c}\cap D_{c^{\prime }}=\emptyset \;\left(c\neq c^{\prime }\right)$$

The main objective of this stage is to determine whether the dataset has a balanced or imbalanced structure by examining the sample counts of the classes. For this purpose, the class sizes $|D_{1}\left|,\ldots ,\right|D_{K}|$ are first calculated, and then the majority and minority classes are identified. If an extremely imbalanced structure is detected, the ratio between the majority and minority classes is evaluated using a predefined threshold to prevent disruption of the subsequent sampling-strategy search process. In this context, when the majority class is denoted by $D_{\mathrm{maj}}$ and the minority class by $D_{\min}$, that is, the largest and the smallest of the *K* class subsets, the preliminary control step is defined by the condition in Equation (2). In this way, the method applies a reduction strategy to mitigate the imbalance in the data.

(2) $$\frac{|D_{\mathrm{maj}}|}{|D_{\min}|}>\tau$$

Here, τ denotes the predefined threshold used to identify severe imbalance, and it is set to 15 in all experiments. If this condition is satisfied, every class whose size exceeds $\tau |D_{\min}|$ is reduced to $\tau |D_{\min}|$ records by random undersampling before proceeding to sampling-strategy selection, while the remaining classes are left unchanged. In the binary case, this affects only the majority class. The new data structure obtained after this operation can be expressed as shown in Equation (3).

(3) $$D^{\prime }=\overset{K}{\underset{c=1}{⋃}}D_{c}^{\prime },\;\left|D_{c}^{\prime }\right|=\min\left(|D_{c}\left|,\;\tau \right|D_{\min}|\right),\;D_{c}^{\prime }\subseteq D_{c}$$

Here, $D_{c}^{\prime }$ represents the controlled reduced form of class *c*. With τ=15, no reduction is required for WUSTL-EHMS-2020, where the ratio between the largest and the smallest class is approximately 7, whereas for CICIoMT2024, where this ratio is approximately 298, the DDoS, DoS, and MQTT classes are reduced to 192,570 records each. After this operation, the dominant effect of the majority class is constrained, and potential bias in the subsequent sampling-search process is reduced. Conversely, if the condition given in Equation (4) is satisfied, the dataset is transferred directly to the sampling-strategy selection stage without any preliminary reduction.

(4) $$\frac{|D_{\mathrm{maj}}|}{|D_{\min}|}\leq \tau$$

### 3.2. Step 2: Sampling Strategy Selection

The second stage constitutes the core component where the adaptive behavior of the proposed method begins to operate in practice. On the dataset preprocessed in the previous step, a sampling search process is carried out to determine the most suitable balancing strategy for the dataset structure. In this step, oversampling methods and undersampling methods are handled as two separate candidate sets. The oversampling-method set is given in Equation (5), while the undersampling-method set is given in Equation (6).

(5) $$\begin{array}{cc} O=\{ & \;\mathrm{SMOTE}\text{-}\mathrm{IPF},\mathrm{SMOTE}\text{-}\mathrm{Tomek},\mathrm{ADASYN},\\ & \;\mathrm{Polynomfit},\mathrm{ProWSyn},\mathrm{Lee},\\ & \;S\text{-}\mathrm{SMOTE},\mathrm{CCR},\mathrm{LVQ}\text{-}\mathrm{SMOTE}\} \end{array}$$

(6) $$\begin{array}{cc} U=\{ & \;\mathrm{none},\mathrm{ENN},\mathrm{IHT},\\ \; & \;\mathrm{NCR},\mathrm{OSS},\mathrm{TomekLinks}\} \end{array}$$

Here, the none element in set *U* represents the case where no undersampling is applied. In this way, the possibility that only oversampling may be sufficient for some datasets is also included in the evaluation process. To determine the most suitable balancing structure specific to the dataset, all possible pairings between *O* and *U* are defined through the Cartesian product:

(7) S=O×U

Each element in set *S* represents a candidate balancing strategy composed of one oversampling method and one undersampling method, as shown in Equation (8).

(8) $$s_{ij}=\left(o_{i},u_{j}\right),\;o_{i}\in O,\;u_{j}\in U$$

With this proposed structure, instead of following a fixed sampling approach, the system can systematically evaluate different oversampling–undersampling pairs that may be suitable for each dataset (the appropriate methods may vary by dataset). Each candidate sampling pair produced by the Cartesian product is separately subjected to training and validation with the classifiers in the base-learner set. The base-learner set is defined in Equation (9).

(9) $$\begin{array}{cc} B=\{ & \;\mathrm{Decision}\;\mathrm{Tree},\mathrm{Extra}\;\mathrm{Tree},\mathrm{Random}\;\mathrm{Forest},\\ & \;\mathrm{LightGBM},\mathrm{CatBoost},\mathrm{XGBoost},\\ & \;\mathrm{Gradient}\;\mathrm{Boost}\} \end{array}$$

Accordingly, each sampling strategy in set *S* is evaluated together with each base learner in set *B*; thus, the effects of both sampling pairs and different model structures on the data are analyzed jointly. The adopted approach ensures that possible interactions between sampling strategies and base learners are taken into account. Therefore, in a given dataset, which balancing pair yields more effective results with which model structure is converted into a dynamically determined selection criterion. All validation results obtained during evaluation are considered as an F1-score-based performance set. Let $s_{ij}\in S$ denote a sampling pair and $b_{k}\in B$ denote a base learner; if the corresponding validation performance is expressed by $f_{ij}^{\left(k\right)}$, all performance values can be defined as in Equation (10).

(10) $$F=\left\{f_{ij}^{\left(k\right)}\;|\;s_{ij}\in S,\;b_{k}\in B\right\}$$

The set F contains the F1 scores obtained from all sampling-pair and base-learner combinations. The main objective is to identify the sampling pair that produces the highest F1 score within F. After evaluating this set, the most successful augmentation strategy is expressed as shown in Equation (11).

(11) $$s^{*}=\left(o^{*},u^{*}\right)\in S$$

Here, $s^{*}$ represents the candidate balancing strategy that yields the maximum F1 value in the performance set. Thus, the selected $s^{*}$ pair is accepted as the final augmentation strategy for the corresponding dataset.

This best sampling pair is then applied to the dataset obtained in the previous step to derive a new data structure. In this process, the selected oversampling method $o^{*}$ is first applied, followed by the selected undersampling method $u^{*}$; consequently, a more balanced and more representative dataset is obtained for subsequent stages. For multi-class data, the oversampling method is applied in a one-versus-many manner, so that each class is oversampled to the size of the largest class in $D^{\prime }$. The undersampling method $u^{*}$ then removes ambiguous or noisy records near the class boundaries, so the final class sizes are close to, but not exactly, equal. As a result, this step in the proposed approach evaluates sampling methods not individually but through the pairs they form with each other and through their behavior under different base learners, thereby adopting an adaptive approach that can conform to the characteristics of each dataset.

### 3.3. Step 3: Dataset Balancing and Base Learner Selection

In the third stage, the best sampling pair identified in the previous step is finalized and used to construct the balanced dataset that will be used throughout the remaining phases of the framework. Let the preprocessed dataset be denoted by $D^{\prime }$, and let the selected sampling pair be $s^{*}=\left(o^{*},u^{*}\right)$. The balanced dataset is obtained by sequentially applying oversampling and undersampling as follows:

(12) $$D_{\mathrm{bal}}=u^{*}\left(o^{*}\left(D^{\prime }\right)\right)$$

In this way, the class distribution is improved while preserving representative decision patterns for both classes.

After constructing $D_{\mathrm{bal}}$, all candidate base learners in set *B* are retrained on this finalized data structure and re-evaluated on the validation subset. Let the resulting F1 score of learner $b_{k}\in B$ be represented by $g_{k}$. The performance set is then expressed as:

(13) $$G=\{g_{k}|b_{k}\in B\}$$

The purpose of this stage is not to keep a single fixed learner, but to identify multiple strong and complementary learners for the stacking architecture. Therefore, the top three base learners with the highest F1 values are selected and defined as:

(14) $$B_{\mathrm{top}3}=\mathrm{Top}3\left(G\right)$$

The selected set $B_{\mathrm{top}3}$ constitutes the final base-learner pool for subsequent feature-selection and meta-learning stages. As a result, the proposed framework preserves its adaptive behavior by selecting base learners according to dataset-specific evidence rather than relying on manually predetermined model choices.

Once $B_{\mathrm{top}3}$ is determined, the selected base learners can be examined with an optional explainability analysis before the final ensemble is constructed. A global SHAP ranking identifies the traffic and telemetry variables that dominate the decisions of each learner, a SHAP beeswarm plot shows the direction and distribution of these effects across instances, and SHAP interaction-value matrices reveal pairwise feature dependencies. Together, these analyses show whether the learners rely on meaningful network and biometric attributes and how their decision logic differs, which informs the model-specific optimization in Step 4.

### 3.4. Step 4: Feature Selection and Construction of the Final Ensemble Structure

**Figure 2:**
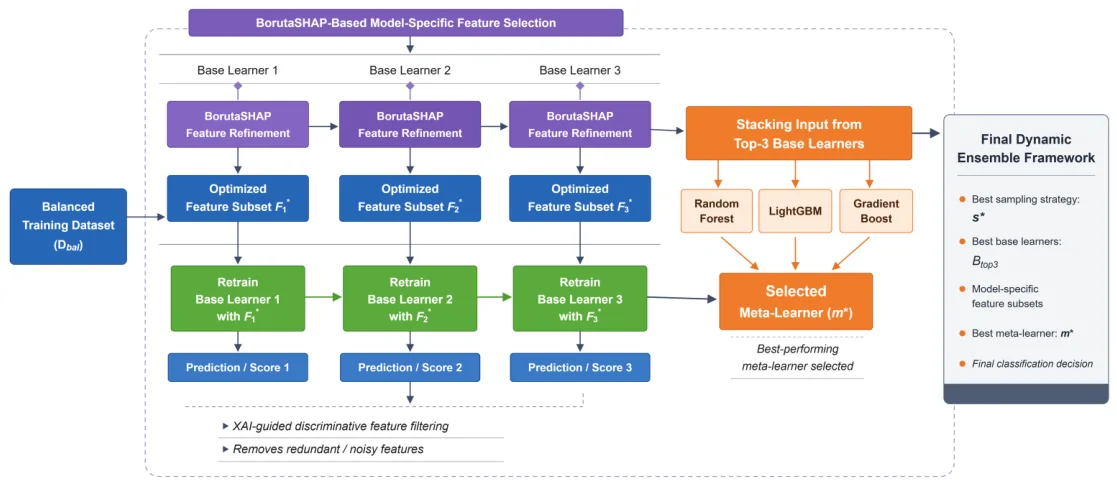
Detailed workflow of Step 4 in the proposed method.

This step takes the three base learners in $B_{\mathrm{top}3}\subset B$, selected on the balanced dataset $D_{\mathrm{bal}}$, as input and covers two tightly coupled processes: identifying the most suitable feature subset for each learner through a comparison of candidate feature-selection methods, and selecting the meta-learner of the final stacking ensemble. Its workflow is shown in Figure 2, and the complete procedure is given in Algorithm 1.

Accordingly, candidate feature-selection methods are evaluated for each base learner in $B_{\mathrm{top}3}$, including RFE, L1/Lasso-based selection, and BorutaSHAP. Rather than fixing a single method in advance, the framework compares these alternatives under the same balanced-data setting and retains the configuration that provides the most suitable performance profile for the corresponding learner. In this way, feature selection becomes a data- and model-adaptive decision stage instead of a static preprocessing choice.

Throughout this paper, L1/Lasso denotes L1-regularized logistic regression rather than Lasso regression. It is fitted with the SAGA solver for the regularization strengths C∈{0.01,0.1,1.0} on features standardized with a scaler fitted on the training data, and a feature is retained when the sum of the absolute values of its coefficients across classes exceeds the default threshold of $10^{-5}$. RFE is implemented as recursive feature elimination with cross-validation, in which the default-parameter version of the corresponding base learner serves as the estimator, one feature is removed at each step, and the number of retained features is determined by five-fold stratified cross-validation using the weighted F1-score. BorutaSHAP uses the same default-parameter base learner with SHAP-based importance and 100 trials, and its accepted features form the selected subset. For each base learner, the configuration with the highest validation F1-score is retained, and ties are resolved in favor of the smaller feature subset.

BorutaSHAP is included as an explainable-AI-driven candidate rather than being assumed to be superior, so the framework keeps it when it is beneficial and prefers RFE or L1/Lasso when they yield better learner-specific results. Each model in $B_{\mathrm{top}3}$ is therefore represented by the subset that best fits its own decision structure, since different learners may extract meaningful patterns from different features. With these learner-specific subsets, each model is retrained and evaluated on the validation subset.

Before ensembling, each base learner is further tuned by Bayesian optimization within model-specific parameter bounds, maximizing the mean F1-score over five stratified folds of the training subset. The optimized setting is used unless the default setting yields a higher validation F1-score. The search spaces of the seven candidate base learners, summarized in Table 2, are chosen to cover the practically relevant operating range of each tree-based and boosting-based model.

**Algorithm 1:** Proposed Dynamic Framework for IoMT Attack Detection

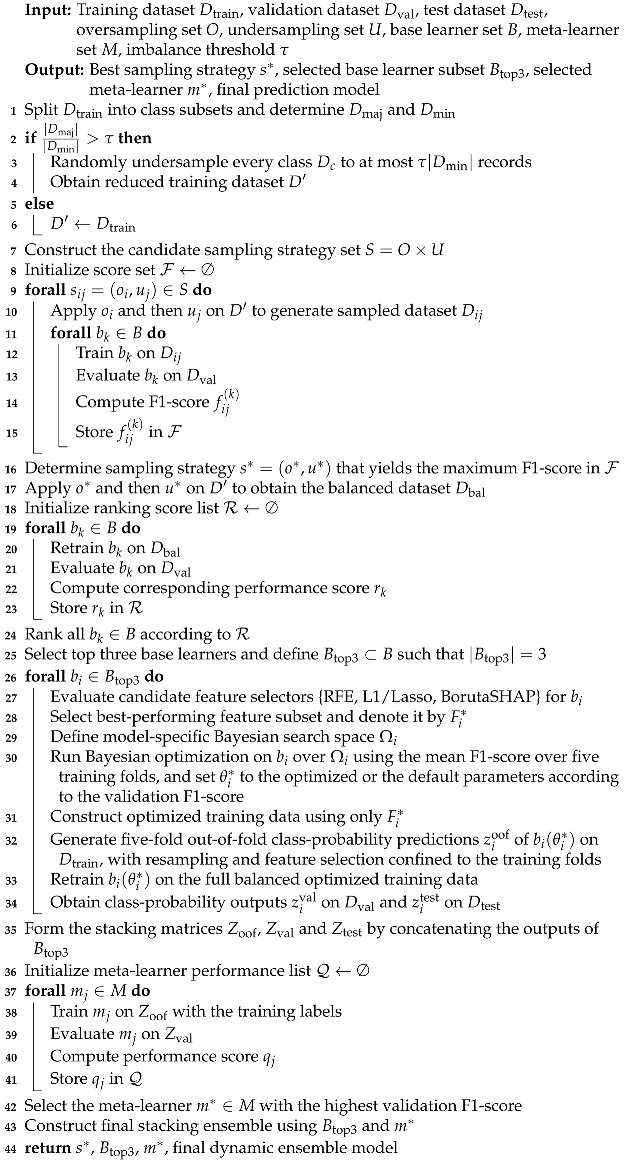



In this way, the stacking layer receives output vectors computed from the model-specific optimized feature representations of the base learners rather than the raw data.

The outputs of the selected three base learners are then combined using a stacking approach. Instead of directly aggregating individual model decisions, the proposed method remaps these decisions through a higher-level learner. In this context, the candidate meta-learner set is defined as {Random Forest,LightGBM,XGBoost,Gradient Boost}. Each candidate is trained on the five-fold out-of-fold class-probability predictions of the optimized base learners in $B_{\mathrm{top}3}$ on the training subset, assembled in the original sample order and concatenated across the base learners, together with the corresponding training labels. During out-of-fold generation, resampling and feature selection are confined to the training folds. The resulting meta-learner candidates are evaluated on the separate validation subset using the F1-score as the selection criterion. The goal of this comparison is to determine the most effective upper-level decision pipeline for the stacking architecture. The candidate with the highest validation F1-score is selected as the meta-learner $m^{*}$. Consequently, the decision outputs of the selected three base learners are fused by this meta-learning layer to produce the system’s final classification decision.

At the end of this step, the sampling strategy, the base learners, their feature subsets and hyperparameters, and the meta-learner have all been determined from the data of the target network, so that data balancing, feature selection, model selection, and decision fusion are optimized as an integrated whole. The complete pseudo-code of the method is provided in Algorithm 1.

## 4. Experimental Results

This section evaluates D3P on two IoMT datasets. For each dataset, the results of the intermediate stages, namely data balancing, feature selection, hyperparameter optimization, and stacking-based decision fusion, are presented first, followed by a comparison of the final architecture with the individual base learners, so that the contribution of each stage can be analyzed.

To evaluate the effectiveness of the proposed method, experimental studies were conducted on two different datasets: WUSTL-EHMS-2020 and CIC IoMT 2024 [48,50]. The WUSTL-EHMS-2020 dataset was developed for Internet of Medical Things (IoMT) cybersecurity research and contains a total of 44 features: 35 network flow metrics, 8 patient biometric attributes, and 1 class label. In this respect, the dataset provides a hybrid structure that includes both network traffic and healthcare-specific biometric information. Since it was derived from the EHMS test environment, it enables assessment of how well methods adapt to realistic healthcare monitoring scenarios [50]. The original dataset is publicly available and was obtained from the official repository at https://www.cse.wustl.edu/~jain/ehms/index.html (accessed on 20 October 2026). The CIC IoMT 2024 dataset is a more recent dataset created to investigate cyberattacks targeting healthcare devices under a multi-protocol structure. According to the official description, the dataset includes 18 different cyberattacks targeting 40 IoMT devices and covers multiple communication protocols commonly used in healthcare devices, including Wi-Fi, MQTT, and Bluetooth [48]. The dataset is publicly available at: https://www.unb.ca/cic/datasets/iomt-dataset-2024.html (accessed on 20 October 2026).

WUSTL-EHMS-2020 and CICIoMT2024 were obtained in January 2025 from the official repositories provided by Washington University in St. Louis and the University of New Brunswick (https://cicresearch.ca/IOTDataset/CICIoMT2024/, accessed on 20 October 2026), respectively. WUSTL-EHMS-2020, which contains 16,318 records without a publisher-defined partition, was randomly divided into training, validation, and test subsets with a 70:10:20 ratio. For CICIoMT2024, the publisher-provided training (7,160,831 records) and test (1,614,182 records) partitions were preserved, and the training partition was further divided into 80% training and 20% validation subsets. The random splits were performed without stratification, and resampling was applied exclusively to the training subsets, whose class distributions before and after balancing are reported in Section 4.1 and Section 4.2. The validation and test subsets were not resampled. Each run used a single random seed for all stochastic stages, including data splitting, resampling, stochastic learners, and Bayesian optimization, and five runs were performed with seeds 0, 1, 7, 42, and 777. The intermediate results reported in Section 4.1 and Section 4.2 are computed on the validation subset of the run with seed 42 as a representative example, whereas the final test results are reported as the mean ± standard deviation over the five runs. The publisher-provided CICIoMT2024 training–test separation, established at the PCAP-file level (51 training and 21 test files), was retained without redistributing records, whereas the additional training–validation split and the WUSTL-EHMS-2020 split were performed at the record level without a device- or session-grouping constraint. Consequently, related records may appear across these subsets.

Before splitting, both datasets were cleaned as follows. From WUSTL-EHMS-2020, the identifier attributes SrcAddr, DstAddr, Sport, Dport, SrcMac, and DstMac were removed, together with Dir, which takes a single constant value, Flgs, a categorical flag field without documentation, and Attack Category, which is a fine-grained version of the class label. The remaining 35 input features comprise 27 network flow metrics, including Packet_num, and 8 patient biometric attributes. All 45 features of CICIoMT2024 were retained because this dataset contains no address or device identifiers. Exact duplicate records were then removed within each source file. Infinite values were converted to missing values, and missing values were imputed with the column median computed separately for each source file before splitting. WUSTL-EHMS-2020 contained no missing or infinite values, so this imputation affected only CICIoMT2024. After these steps, all input features are numeric, so no categorical feature encoding was required, and the class labels were label-encoded using an encoder fitted on the training subset. No feature scaling was applied to the inputs of the learners. The L1-regularized logistic regression used for feature selection standardizes the features internally, with the scaler fitted on the training data only. The preliminary majority-class reduction (imbalance threshold τ=15) and the selected resampling strategy were applied to the training subset only. Apart from the file-level imputation, every fitted transformation and sampling operation used the training subset exclusively.

Exact and near-identical overlap between the partitions was also quantified on the split of the run with seed 42. No identical feature vectors occur within or between the WUSTL-EHMS-2020 partitions. In CICIoMT2024, preprocessing removed 7184 duplicate records (5119 from the training partition and 2065 from the test partition), after which 195 of the 1,612,117 test records (0.0121%) have an identical feature vector in the training partition. Near-identical overlap was measured using Euclidean nearest-neighbor distances in the feature space standardized on the training partition. For WUSTL-EHMS-2020, 3.19% of the test records have a training record within a distance of 0.01, and this proportion depends on the distance threshold and the feature representation. In repeated comparisons with equal-sized reference and query sets, the proportions for test, validation, and disjoint training queries were of a similar order of magnitude (2.56%, 2.60%, and 2.13%). The Packet_num attribute of WUSTL-EHMS-2020 follows the record order of the dataset almost exactly (Spearman correlation above 0.9999) and therefore acts as a capture-order index. Because attack records occur in 88 contiguous segments with a median length of 24 records, this attribute can carry temporal information under a random record-level split. When Packet_num, which otherwise separates many near-identical records, is excluded, the proportion of WUSTL-EHMS-2020 test records with a training record within a distance of 0.01 increases to 15.17%. Repeating the complete D3P pipeline without this attribute, including sampling-strategy selection, feature selection, hyperparameter optimization, and stacking, over the same five seeds yields 94.91 ± 0.17% accuracy and 94.56 ± 0.17% F1-score on the test set (Section 5.1). For CICIoMT2024, 45.0% of the test records have a near-identical training record, compared with 43.7% for held-out training records matched against the remaining training data. Because these two comparisons use reference sets of different sizes, they are interpreted descriptively. Generalization to independent recording sessions, unseen devices, and chronologically separated traffic has not been evaluated and remains a limitation of this study and a direction for future work.

The performance of the proposed method was evaluated using the commonly used classification metrics of accuracy, precision, recall, and F1-score. When considered together, these metrics reveal not only the overall correct classification rate, but also how accurately the attack class is detected and how the false positive/false negative balance is shaped. Especially in imbalanced data structures, focusing only on accuracy may be misleading; therefore, complementary metrics such as precision, recall, and F1-score are reported jointly in this study. The evaluation metrics used in this study are defined as follows:

(15) $$Accuracy=\frac{TP+TN}{TP+TN+FP+FN}$$

(16) $$Precision=\frac{TP}{TP+FP}$$

(17) $$Recall=\frac{TP}{TP+FN}$$

(18) $$F1\text{-}score=\frac{2\times Precision\times Recall}{Precision+Recall}$$

Here, TP denotes true positives, TN true negatives, FP false positives, and FN false negatives. While the accuracy metric expresses overall classification performance, precision indicates how many samples predicted as attacks are truly attacks. Recall indicates how many real attack samples are correctly detected. The F1-score provides a more balanced performance assessment, particularly on imbalanced datasets, by taking the harmonic mean of precision and recall.

### 4.1. Results on WUSTL-EHMS-2020

Table 3 reports the class distribution of the WUSTL-EHMS-2020 training subset before and after balancing. The original training data are clearly imbalanced, with 9990 benign and only 1432 attack samples.

Such a distribution can cause the majority class to dominate training and prevent sufficient representation of the attack class. After the sampling search, the selected IHT-CCR strategy produced 9800 benign and 9794 attack samples. This near-symmetric distribution, obtained by increasing the attack class and moderately reducing the benign class, provides a more suitable basis for the subsequent stages. The effect of balancing on the base learners is shown in Table 4, which compares each learner trained on the original and on the balanced training data.

The base learner performances obtained on the original and balanced training data are presented in Table 4. As observed in the table, data balancing improved classification performance for most of the models. In particular, the F1-score increased from 0.9551 to 0.9605 for XGBoost, from 0.9510 to 0.9616 for CatBoost, and from 0.9517 to 0.9564 for LightGBM. These gains indicate that the IHT-CCR-based sampling strategy, which was automatically selected by the proposed method for this specific dataset, provides a more pronounced contribution particularly for strong boosting-based learners.

For the remaining models, the effect is mixed: Decision Tree changes from 0.9441 to 0.9449, Random Forest from 0.9255 to 0.9253, Extra Trees from 0.9222 to 0.9189, and GradientBoost from 0.9471 to 0.9511. This indicates that the balancing process does not produce identical effects across all models and can lead to architecture-dependent outcomes.

A natural question is whether these gains reflect a genuine improvement in detection or merely a side effect of balancing on the metrics. The evaluation design rules out the latter, because balancing is applied only to the training subset, whereas the validation subset on which Table 4 is computed keeps its original class distribution. The scores before and after balancing are therefore measured on exactly the same imbalanced data, and the differences reflect how well each learner generalizes to the real class distribution rather than a change in the evaluation set. Moreover, because the metrics are weighted by class support, they would penalize any substantial loss on the majority class, so the gains of XGBoost, LightGBM, CatBoost, and GradientBoost indicate that the better representation of the attack class was achieved without a substantial loss on benign traffic. The nearly unchanged scores of Random Forest and Extra Trees indicate that the size of this benefit depends on the learner, which is precisely why D3P selects the sampling strategy and the base learners jointly instead of applying a fixed balancing step.

Based on these results, XGBoost, CatBoost, and LightGBM, which produced the highest F1-scores on the balanced data, were selected by the framework as the base learners for the subsequent stages.

Since the selected learners may not benefit equally from the same feature subsets, feature selection is treated as a model-specific optimization step rather than a fixed preprocessing choice.

Feature selection results obtained for the three strongest base learners on the balanced WUSTL-EHMS-2020 dataset are presented in Table 5. As can be seen, feature selection produces different outcomes across models. For XGBoost, the highest F1-score is obtained with all features (0.9610), while BorutaSHAP, RFE, and Lasso (C: 0.01) yield 0.9563, 0.9589, and 0.9567, respectively. For CatBoost, the strongest result is produced by Lasso (C: 0.1) with an F1-score of 0.9607, followed closely by RFE (0.9606), whereas all features and BorutaSHAP yield 0.9602 and 0.9598. For LightGBM, all features and RFE provide the same highest F1-score (0.9589), while BorutaSHAP and Lasso (C: 0.01) remain at 0.9563 and 0.9567. Thus, even under the same dataset and the same candidate model group, the effect of feature selection methods clearly depends on the structure of the base learner.

Table 5 also shows that BorutaSHAP does not yield the highest F1-score for every model. Its value lies in combining SHAP-based importance with the shadow-feature test of Boruta, which compares each feature with randomized copies and thus filters out features whose high importance is not reliable. The framework therefore treats BorutaSHAP as one of the candidate selectors and retains it only when it yields the best validation performance for a learner. The feature subsets used by the selected configuration of each base learner are listed in Appendix A (Table A1).

Table 6 compares the selected base learners under default and Bayesian-optimized hyperparameters after feature selection. Optimization improves all three models, increasing the F1-score from 0.9607 to 0.9713 for CatBoost and from 0.9589 to 0.9676 for LightGBM, and slightly from 0.9688 to 0.9690 for XGBoost, which indicates that suitable hyperparameters help each boosting-based learner form more effective decision boundaries.

The resulting hyperparameter configurations are reported in Appendix A (Table A2).

**Figure 3:**
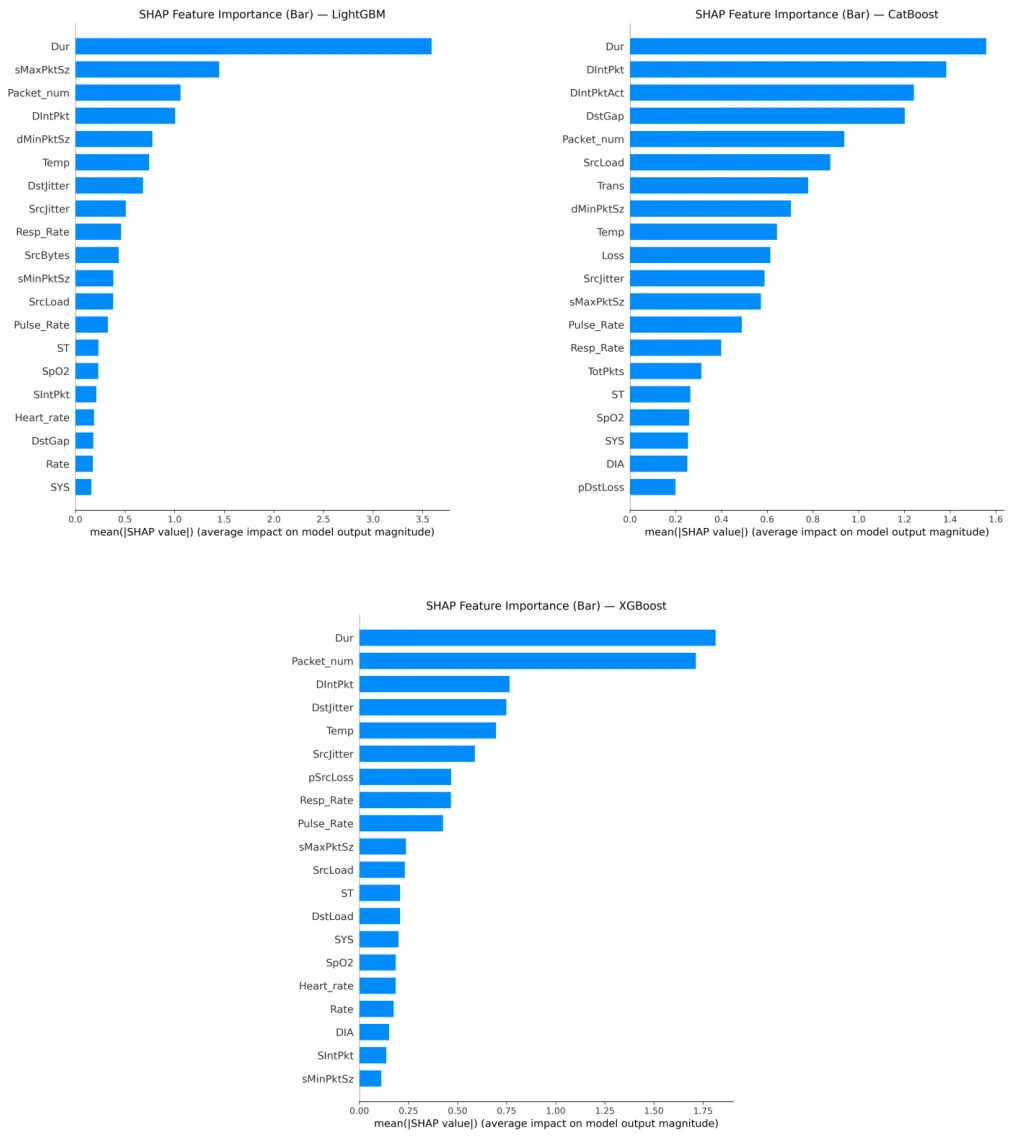
Comparative global SHAP feature-importance rankings for optimized LightGBM, CatBoost, and XGBoost base learners on the WUSTL-EHMS-2020 dataset.

**Figure A1:**
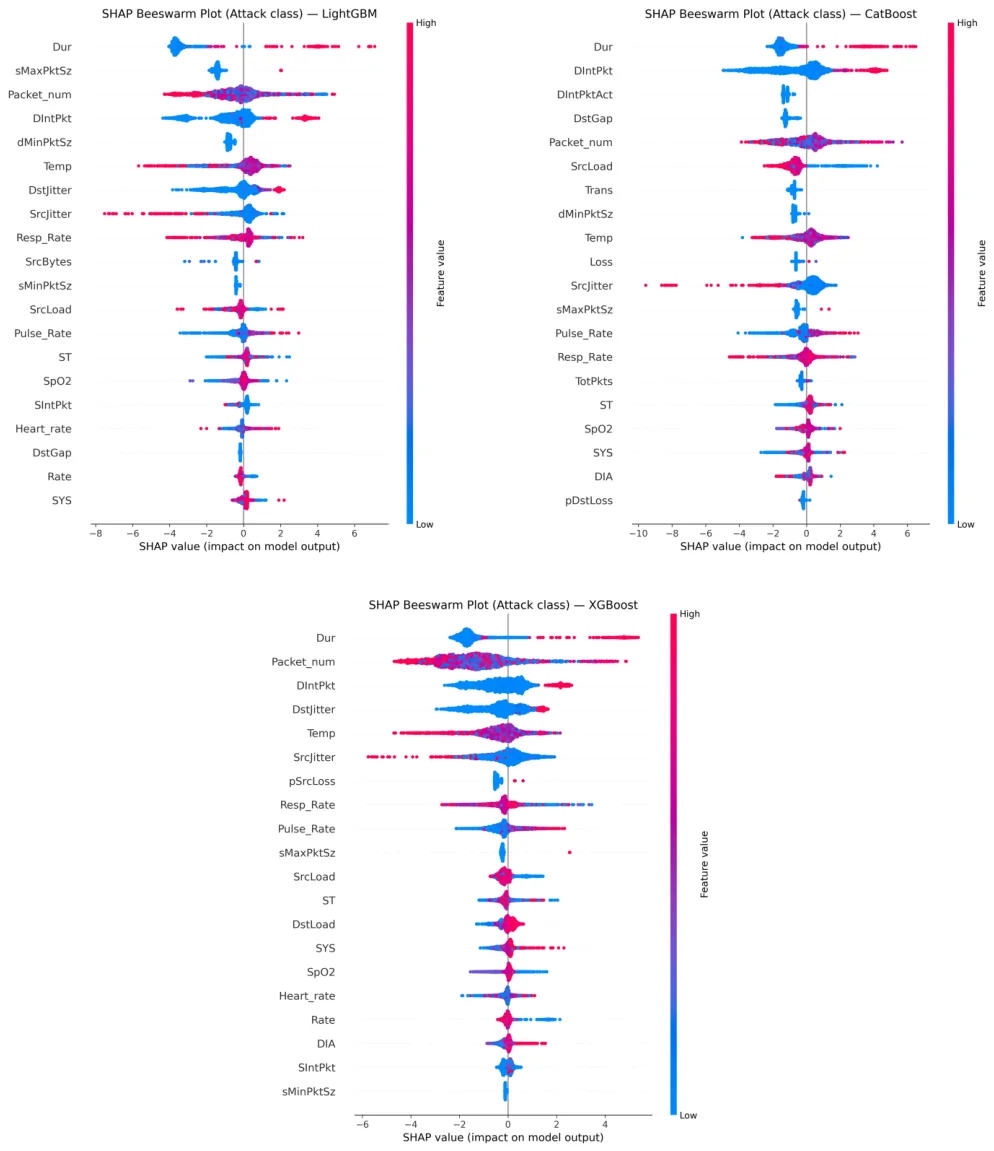
Global SHAP beeswarm comparison for optimized LightGBM, CatBoost, and XGBoost base learners on WUSTL-EHMS-2020.

**Figure A2:**
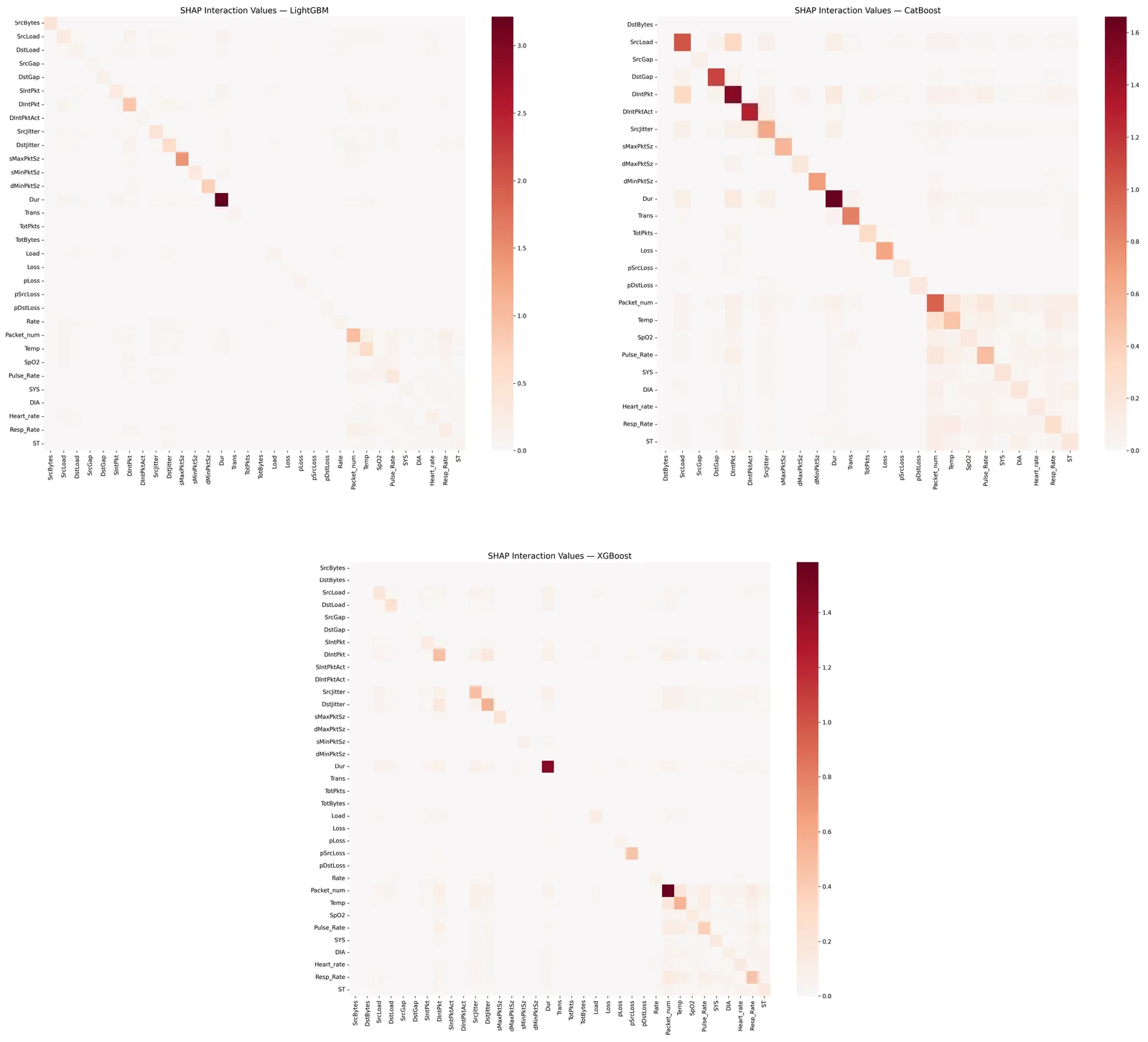
SHAP interaction-value matrix comparison for optimized LightGBM, CatBoost, and XGBoost base learners on WUSTL-EHMS-2020.

Figure 3 compares the global SHAP feature-importance rankings of the three optimized base learners. In all three models, temporal and packet-level traffic characteristics dominate, with Dur ranked first and DIntPkt and Packet_num among the most influential variables, while biometric variables such as Temp, Pulse_Rate, and SpO2 also remain among the top features. Attack discrimination in WUSTL-EHMS-2020 is therefore driven by flow duration, inter-packet timing behavior, and packet-structure dynamics combined with physiological context rather than by a single isolated signal. The signed SHAP-value distributions and the pairwise SHAP interaction values of the three learners are provided in Appendix B (Figure A1 and Figure A2). They show that the three learners prioritize and combine the features differently, which supports the use of stacking to exploit their complementary decision logic.

Table 7 compares the meta-learner candidates of the stacking stage. The RandomForest meta-learner achieves the highest validation scores, with 0.9782 accuracy, 0.9779 precision, 0.9782 recall, and a 0.9780 F1-score, followed by the LightGBM, XGBoost, and GradientBoost meta-learners with F1-scores of 0.9759, 0.9753, and 0.9746, respectively. This shows that the choice of the meta-learner also has a direct impact on the final performance of the stacking architecture.

**Figure 4:**
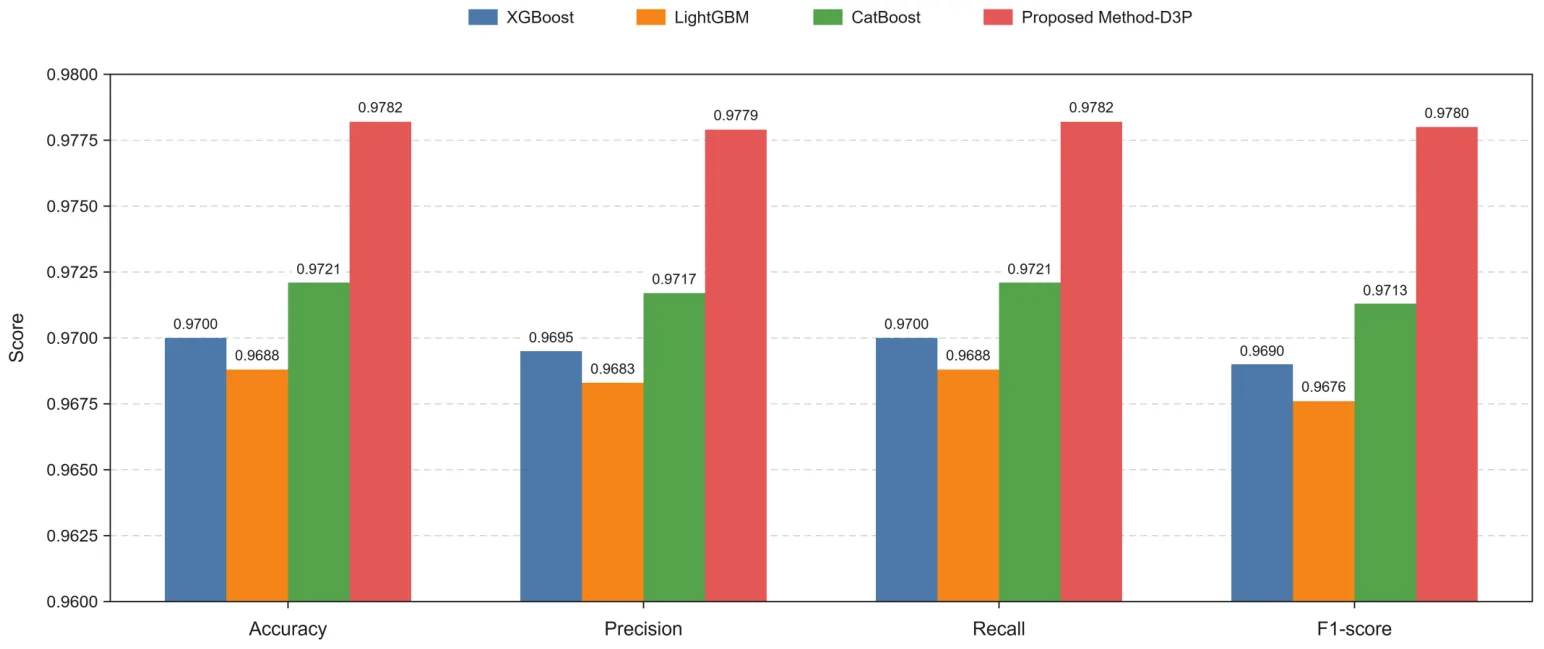
Comparison of the optimized base learners and the final proposed dynamic stacking system (Dynamic Data-Driven Pipeline D3P) on the WUSTL-EHMS-2020 validation subset.

Figure 4 compares the optimized base learners with the final system on the validation subset. D3P with the RandomForest meta-learner outperforms all individual base learners across all evaluation metrics, and its validation F1-score of 0.9780 exceeds that of CatBoost (0.9713), the strongest optimized base learner. This indicates that the stacking stage learns complementary information among the base-learner decisions rather than merely aggregating them, which supports the core design rationale of the framework.

For both datasets, precision, recall, and F1-score are computed for each class using Equations (16)–(18), with the respective class treated as the positive class, and are then averaged with weights proportional to the number of samples in each class. Table 8 complements these weighted scores with the per-class results, the macro scores, which give equal weight to each class, and the confusion matrix of D3P on the WUSTL-EHMS-2020 test set for the run with seed 42, one of the five runs summarized in Section 5.1. For the attack class, D3P reaches a precision of 0.9109, a recall of 0.8504, and an F1-score of 0.8796, while the benign class reaches 0.9780, 0.9877, and 0.9828, respectively. The macro F1-score is 0.9312, and the balanced accuracy, defined as the macro recall, is 0.9190. Both are lower than the weighted F1-score of 0.9695, because the attack class, which accounts for 12.9% of the test records, receives the same weight as the benign class in these measures. Of the 421 attack records, 358 are detected and 63 are classified as benign, while 35 of the 2842 benign records are classified as attacks.

### 4.2. Results on CIC IoMT 2024

CICIoMT2024 combines IoMT-specific network traffic with multiple heterogeneous attack types and therefore allows the method to be evaluated under realistic conditions.

Table 9 reports the class distribution of the CICIoMT2024 training subset before and after balancing. The original training subset is highly imbalanced, with 3,823,350 DDoS samples but only 12,838 Spoofing samples, whereas after balancing all classes contain between 190K and 193K samples.

Without balancing, the models would tend to prioritize majority classes such as DDoS and DoS, so that the patterns of underrepresented classes such as Spoofing and Recon might not be learned sufficiently, and these classes could suffer from low recall even when the overall accuracy is high. Since missed detections of Spoofing, Recon, or MQTT-based attacks pose significant security risks in IoMT environments, the balancing step is an essential part of the detection framework for this dataset.

As shown in Table 10, the performances of base learners evaluated on the original and balanced datasets are affected differently across model families. The values shown in bold indicate the results that outperform those obtained on the original dataset, and the comparison is presented on a model-by-model basis. On the original dataset, Decision Tree and Random Forest obtain the highest results, with values close to 0.998 across all four metrics. After data balancing, only a very limited decrease is observed for Decision Tree, whereas clear performance gains emerge particularly for CatBoost, LightGBM, GradientBoost, and, to a lesser extent, XGBoost. This pattern indicates that class imbalance has a negative impact on specific learners and that, after balancing, models can learn more representative decision boundaries. As in Section 4.1, both settings are evaluated on the same validation subset with its original class distribution, so these gains are not a consequence of a rebalanced evaluation set.

On the balanced data, CatBoost becomes the strongest model, reaching 0.9982 in all metrics, while Random Forest remains stable at around 0.9981 in both settings. Based on these results, the framework selected Decision Tree, Random Forest, and CatBoost as the three base learners for the subsequent stages.

To evaluate the contribution of feature selection on CICIoMT2024, Table 11 reports the model-wise comparison across All Features, RFE, BorutaSHAP, and L1/Lasso configurations for the three selected base learners.

Table 11 shows that, at this already high performance level, feature selection acts mainly as a refinement step, but the most suitable method still differs between learners. For Decision Tree, All Features, RFE, and L1/Lasso reach the same F1-score of 0.9977, and RFE is selected because it uses only 8 of the 45 features. For Random Forest, L1/Lasso (C: 0.01) provides the best results for all metrics (0.9982). For CatBoost, L1/Lasso and BorutaSHAP share the same F1-score of 0.9982, and BorutaSHAP is selected because it uses fewer features (32/45). The final choices are therefore RFE for Decision Tree, L1/Lasso for Random Forest, and BorutaSHAP for CatBoost.

The selected feature subsets, listed in Appendix A (Table A3), differ strongly in size, from 8 features for Decision Tree to 42 for Random Forest, but all three share Rate, syn_flag_number, ack_flag_number, ack_count, rst_count, Max, and IAT. This indicates that flow-rate properties, TCP flag operations, and timing features are the key attributes for distinguishing attacks in CICIoMT2024, while each learner uses them at a different level of granularity.

Table 12 compares the selected base learners under default and optimized hyperparameters. Since the baseline performance is already very high, the effect of optimization is model-specific. It lowers the F1-score of CatBoost from 0.9982 to 0.9953, raises that of Decision Tree from 0.9977 to 0.9981, and leaves that of Random Forest unchanged at 0.9982. Optimization is therefore applied selectively for each learner.

The final hyperparameter settings are reported in Appendix A (Table A4). Decision Tree uses its optimized values, while Random Forest and CatBoost are kept at their default settings.

**Figure 5:**
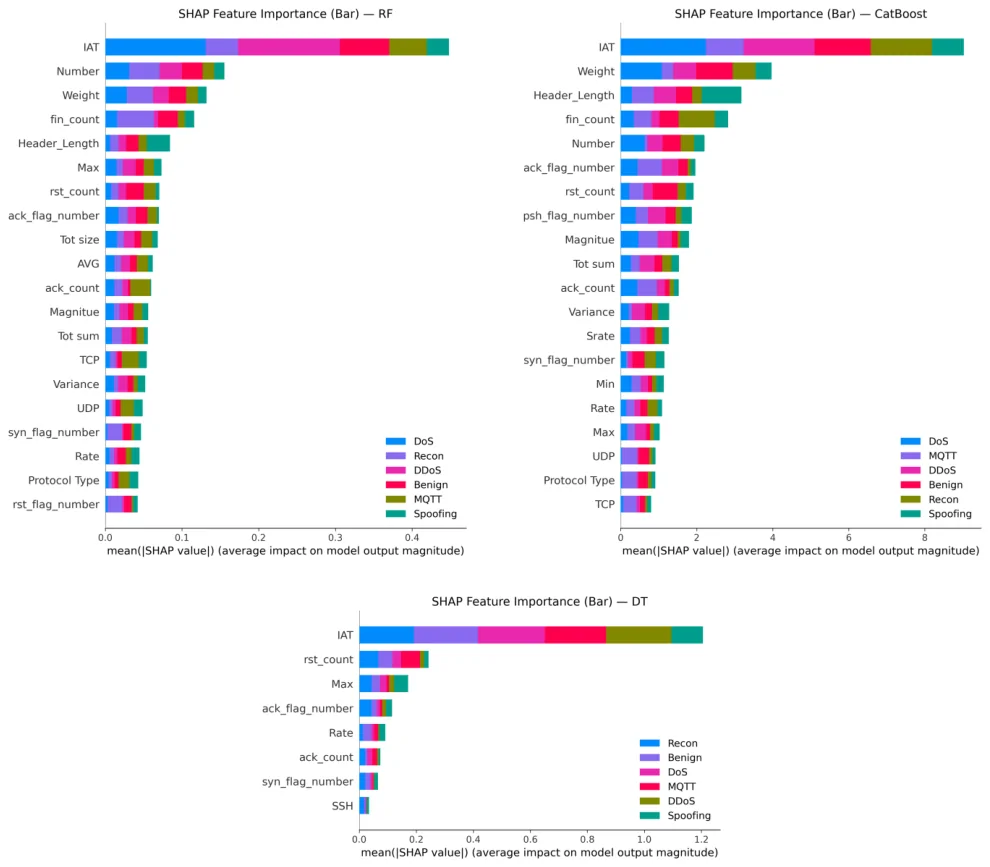
Class-wise global SHAP feature-importance comparison for Decision Tree, Random Forest, and CatBoost on CICIoMT2024.

**Figure A3:**
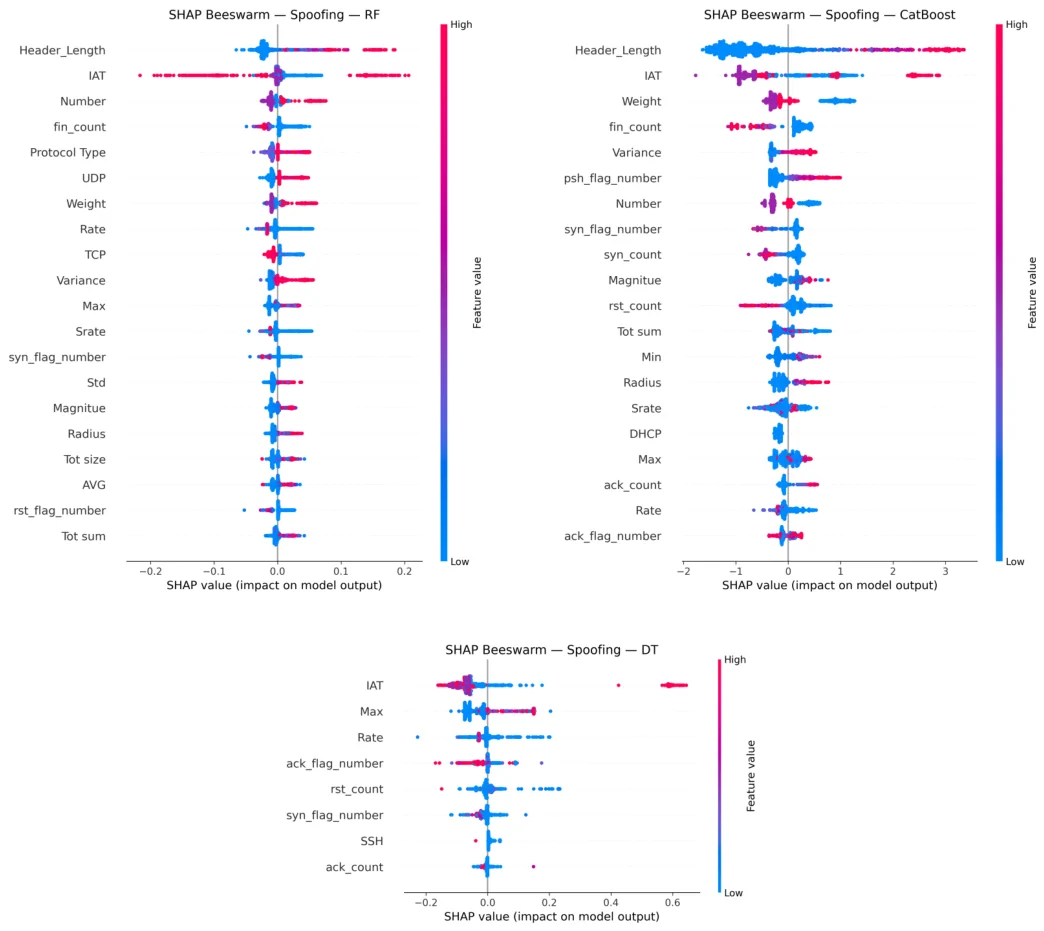
SHAP beeswarm comparison for the Spoofing class on CICIoMT2024 using Decision Tree, Random Forest, and CatBoost.

Figure 5 presents class-wise global SHAP feature-importance plots for the three base learners. In all of them, IAT is the dominant feature, and its importance is shared across multiple classes, which indicates that temporal spacing is the strongest common signal for separating benign and malicious traffic in CICIoMT2024. Features with long bars and a balanced distribution across classes, such as IAT, Number, Header_Length, and selected flag and count variables, act as cross-class discriminators. Random Forest and CatBoost rely on larger sets of packet, header, and flag statistics, whereas Decision Tree concentrates on a few features such as IAT, rst_count, Max, and Rate, so the learners capture complementary class information for the stacking stage. SHAP beeswarm plots for the Spoofing class are provided in Appendix B (Figure A3).

Table 13 compares the meta-learner candidates. Since the base learners already perform at a very high level, the role of the meta-learner is to model the remaining small differences among strong predictions rather than to correct large errors. The Random Forest meta-learner provides the highest and most balanced validation performance (Acc./Prec./Rec./F1 = 0.9985). XGBoost reaches the same F1-score with slightly lower accuracy and recall (0.9984), and LightGBM and GradientBoost reach an F1-score of 0.9984, so Random Forest, which yields the most consistent metric profile, is selected as the final meta-learner.

Compared with the strongest individual base-learner performance in Table 12 (an F1-score of 0.9982), the D3P architecture with the Random Forest meta-learner reaches a validation F1-score of 0.9985 and improves validation performance. Although this increase appears limited in absolute terms, such an improvement is meaningful on a dataset like CIC IoMT 2024 where baseline performance is already very high.

## 5. Discussion

This section places the results of D3P in the context of previous work and examines the scope of the findings. Section 5.1 compares the test results of D3P with those of studies that report results on WUSTL-EHMS-2020 and CICIoMT2024, and Section 5.2 discusses to what extent the findings obtained on these two datasets can be expected to generalize.

### 5.1. Cross-Study Comparison on CICIoMT2024 and WUSTL-EHMS-2020 Datasets

To position the proposed framework against prior studies, Table 14 summarizes reported results on the WUSTL-EHMS-2020 dataset, and Table 15 presents representative results on the CICIoMT2024 dataset. To avoid duplicate references in this manuscript, studies already cited in the bibliography are referenced with their existing keys (e.g., Naeem et al. and Akar et al.), while newly introduced studies are added with dedicated citation keys.

Table 14 shows that the proposed D3P model achieves 97.01% accuracy, 96.94% precision, 97.01% recall, and 96.97% F1-score on the test set of WUSTL-EHMS-2020. Aljuhani et al. used a PSO-DT based explainable IDS and reported 96.57% accuracy with 98.02% F1; compared with that study, our accuracy is higher while their F1 is slightly higher. Kilincer et al. used an MLP model and reported 96.20% accuracy and 96.17% F1, which remains below our D3P scores on all four metrics. Liu used a BiGRU/RBWK architecture and reported 95.60% accuracy and 96.3% F1, again lower than our model across the reported metrics.

Hady et al. evaluated RF, KNN, SVM, and DNN baselines and reported accuracy values between 90.04% and 92.40%; D3P exceeds each of these settings by a clear margin. Zhang et al. reported XGB, RF, KNN, and SVM results, with the strongest configuration reaching 95.01% accuracy and 94.64% F1, which is still below our 97.01% accuracy and 96.97% F1. Pinar et al. tested KNN, SVM, XGB, and DNN variants, where the best result was 96.37% accuracy and 91.30% F1; our model outperforms this best case as well. At the upper end of the table, Nasayreh et al. (99.90% across all metrics) and Zukaib et al. (around 99.5–99.57%) remain above our current WUSTL result, so our method should be positioned as a strong and balanced approach that surpasses many prior papers, though not the absolute top score in every comparison. When the capture-order attribute Packet_num is excluded, D3P achieves 94.91% accuracy and 94.56% F1-score (Table 14). Under this setting, D3P still exceeds the reported results of Hady et al., Dina et al., Naeem et al., and Kumari et al., but falls below those of Aljuhani et al., Ravi et al., Kilincer et al., Liu, Zhang et al., Zukaib et al., and Nasayreh et al. Relative to Pinar et al., its accuracy is lower but its F1-score is higher. Because the feature sets used in prior studies are not always reported, the two D3P rows indicate the performance range of D3P under the two feature settings.

According to Table 15, D3P achieves 99.78%, 99.77%, 99.78%, and 99.77% on accuracy, precision, recall, and F1-score, respectively, in the 6-class CICIoMT2024 setting, outperforming other directly comparable 6-class papers in the table, including Hossain et al. (best 6-class: 98.11% accuracy, 97.88% F1), Akar et al. (6-class LSTM: 98.00%), Alabbadi and Bajaber (X-FuseRLSTM: 98.05%), and Naeem et al. (6-class: 99.00% accuracy). These comparisons show that, under the same class granularity, our method delivers the strongest overall metric profile among the listed studies.

Moreover, there are many binary and mixed setting results with extremely high values like the work by Akar et al. (100% in binary), the work by Akhi et al. (99.90–100% in binary), and the work by Shebl et al. (99.98% in binary, 99.86% in multiclass). It implies that our approach does not always provide the best performance on different task definitions. However, it can be claimed that the key feature of the proposed model lies in achieving the best result on the 6-class setting as an equivalent setting for methodological verification.

Another issue that can be revealed based on the analysis of results from other papers is the lack of metrics reporting in some studies (e.g., N/R). Such an approach reduces the possibility of strict verification of results. On the contrary, in our case, D3P reports consistently high values for all four metrics (Acc./Prec./Rec./F1 = 99.78%/99.77%/99.78%/99.77%). Summarizing the information above about cross-paper comparison with other approaches, the results presented in Table 15 suggest that our approach belongs to the strongest 6-class CICIoMT2024 solutions recently introduced in related papers.

### 5.2. Generalizability of the Proposed Framework

Since the experiments cover two datasets, the extent to which the findings generalize has to be considered separately for the methodology and for the reported performance. At the level of the methodology, D3P does not fix the sampling strategy, the base learners, the feature subsets, the hyperparameters, or the meta-learner in advance, but derives all of them from the training and validation data of the target network. The two datasets illustrate this behavior well, because they differ in nearly every respect. WUSTL-EHMS-2020 is a small binary dataset that combines network and biometric features with a moderate class ratio of approximately 7, whereas CICIoMT2024 is a large six-class, multi-protocol dataset whose largest and smallest classes differ by a factor of approximately 298. For these two datasets, the framework selected different sampling pairs (IHT with CCR, and SMOTE-Tomek with LVQ-SMOTE), different base learners (XGBoost, LightGBM, and CatBoost, and Decision Tree, Random Forest, and CatBoost), and different feature-selection methods for each learner, without any manual redesign of the pipeline. This indicates that the procedure itself transfers to a new labeled dataset.

At the level of the reported performance, the scores are specific to the evaluated datasets and partitioning protocols and should be verified again whenever the framework is applied to a new environment. Both datasets were collected in controlled testbeds, and, as discussed in Section 4, the evaluations do not establish independence across recording sessions, devices, or time. The behavior of D3P on traffic from operational healthcare networks, on previously unseen device types, and under changing traffic patterns therefore remains to be established, and evaluating the framework on additional IoMT datasets is an important direction for future work.

## 6. Conclusions

Due to heterogeneous device structures, class imbalance, variable traffic patterns, and strict explainability requirements, detecting potential attacks in IoMT environments remains a major challenge in the literature. To address these issues, this study proposes Dynamic Data-Driven Pipeline (D3P), an adaptable and explainable intrusion detection framework that can be tailored to different data structures and heterogeneous network contexts. The proposed approach integrates dataset-specific sampling strategy selection, identification of the most suitable base learners, model-specific feature selection, hyperparameter optimization, and stacking-based decision fusion within a unified architecture. In addition, SHAP-, LIME-, and BorutaSHAP-based explainability components are used to support interpretable model decisions.

The experimental findings show that D3P provides balanced performance across two different IoMT datasets. The method achieves a test F1-score of 96.97 ± 0.14% on WUSTL-EHMS-2020, while reaching an F1-score of 99.77 ± 0.05% in the 6-class scenario of CICIoMT2024. These results indicate that the proposed framework delivers a holistic IDS structure that adapts to dataset characteristics while maintaining explainable decision-making. In conclusion, this work presents an application-oriented framework for IoMT security that jointly evaluates data balancing, feature optimization, explainable artificial intelligence, and ensemble learning in an integrated manner. Future work will focus on evaluating the proposed architecture on additional IoMT datasets, adapting it to real-time intrusion detection environments, and extending it to distributed/federated learning scenarios.

## Figures and Tables

**Table 1:** Methodological comparison of recent studies and the proposed D3P framework across five criteria. A check mark denotes that the criterion is satisfied and a cross that it is not.

Paper (Reference)	Data Adaptivity	Explainability	Imbalance Handling	Cross-Dataset Validation	Reproducibility
Aljuhani et al. [5]	✓	✓	×	×	×
Pinar et al. [20]	✓	×	×	✓	✓
Ravi et al. [23]	✓	×	✓	×	×
Naeem et al. [25]	✓	✓	✓	✓	×
Akar et al. [27]	×	×	✓	×	✓
Zukaib et al. [28]	✓	×	✓	✓	✓
Sharma and Shambharkar [30]	×	✓	×	×	✓
Alabbadi and Bajaber [31]	×	✓	✓	✓	✓
Hossain et al. [32]	✓	×	✓	×	✓
Shaikh et al. [33]	✓	×	✓	×	×
Hnamte et al. [34]	×	×	✓	✓	×
Hernandez-Jaimes et al. [35]	✓	×	×	✓	✓
Gueriani et al. [36]	×	×	✓	✓	×
Choubey et al. [37]	×	×	×	×	✓
Akhi et al. [38]	×	×	✓	✓	✓
Alturki and Alsulami [39]	✓	×	✓	✓	✓
Shebl et al. [40]	×	×	✓	✓	✓
Jeremiah et al. [41]	✓	×	✓	×	✓
Hady et al. [42]	×	×	×	×	×
Dina et al. [43]	×	×	✓	✓	✓
Zhang et al. [44]	×	×	✓	✓	×
Kumari et al. [45]	✓	×	✓	×	×
Liu [46]	✓	×	✓	✓	✓
Nasayreh et al. [47]	✓	×	✓	✓	✓
Dadkhah et al. [48]	×	×	×	×	✓
**Ours (D3P)**	✓	✓	✓	✓	✓

**Table 2:** Bayesian optimization search spaces for candidate base learners.

Model	Hyperparameter	Search Range
DT	max_depth	[1, 30]
	min_samples_split	[2, 20]
	min_samples_leaf	[1, 20]
	max_features	[0.1, 1.0]
RF	n_estimators	[50, 500]
	max_depth	[1, 40]
	min_samples_split	[2, 20]
	min_samples_leaf	[1, 20]
	max_features	[0.1, 1.0]
ExtraTrees	n_estimators	[50, 500]
	max_depth	[1, 40]
	min_samples_split	[2, 20]
	min_samples_leaf	[1, 20]
	max_features	[0.1, 1.0]
XGBoost	n_estimators	[50, 500]
	max_depth	[1, 20]
	learning_rate	[0.01, 0.3]
	subsample	[0.5, 1.0]
	colsample_bytree	[0.5, 1.0]
	min_child_weight	[1, 20]
	reg_lambda	[0.1, 10]
LightGBM	n_estimators	[50, 500]
	learning_rate	[0.01, 0.3]
	num_leaves	[10, 200]
	min_child_samples	[5, 100]
	subsample	[0.5, 1.0]
	colsample_bytree	[0.5, 1.0]
CatBoost	iterations	[50, 500]
	depth	[3, 10]
	learning_rate	[0.01, 0.3]
	l2_leaf_reg	[0.1, 10]
	border_count	[32, 255]
GradientBoost	max_iter	[50, 500]
	max_depth	[1, 20]
	learning_rate	[0.01, 0.3]
	min_samples_leaf	[5, 50]
	l2_regularization	[0, 10]

**Table 3:** Train set statistics after balancing for the WUSTL-EHMS-2020 dataset.

Class	Original Train Set	Balanced Train Set
Benign	9990	9800
Attack	1432	9794

**Table 4:** Performance comparison of base learners on the original and balanced WUSTL-EHMS-2020 dataset. Results are reported on the validation subset. For each model, the better of the two compared settings is shown in bold.

Base Learner	Original WUSTL-EHMS-2020				Balanced (IHT-CCR) WUSTL-EHMS-2020			
	Accuracy	Precision	Recall	F1-Score	Accuracy	Precision	Recall	F1-Score
Decision Tree	0.9442	0.9439	0.9442	0.9441	**0.9449**	**0.9450**	**0.9449**	**0.9449**
Random Forest	**0.9347**	**0.9372**	**0.9347**	**0.9255**	0.9347	0.9375	0.9347	0.9253
Extra Trees	**0.9320**	**0.9334**	**0.9320**	**0.9222**	0.9289	0.9287	0.9289	0.9189
XGBoost	0.9577	0.9573	0.9577	0.9551	**0.9626**	**0.9624**	**0.9626**	**0.9605**
LightGBM	0.9550	0.9549	0.9550	0.9517	**0.9593**	**0.9595**	**0.9593**	**0.9564**
CatBoost	0.9547	0.9553	0.9547	0.9510	**0.9638**	**0.9643**	**0.9638**	**0.9616**
GradientBoost	0.9510	0.9507	0.9510	0.9471	**0.9547**	**0.9550**	**0.9547**	**0.9511**

**Table 5:** Feature selection comparison for the top-performing base learners on the balanced WUSTL-EHMS-2020 dataset. Results are reported on the validation subset. The configuration selected for each base learner is shown in bold.

Base Learner	Feature Selection Method	Accuracy	Precision	Recall	F1-Score
XGBoost	**All Features (35/35)**	**0.9629**	**0.9626**	**0.9629**	**0.9610**
	BorutaSHAP (15/35)	0.9593	0.9598	0.9593	0.9563
	RFE (32/35)	0.9614	0.9616	0.9614	0.9589
	Lasso (C: 0.01) (16/35)	0.9596	0.9600	0.9596	0.9567
CatBoost	All Features (35/35)	0.9626	0.9632	0.9626	0.9602
	BorutaSHAP (32/35)	0.9623	0.9629	0.9623	0.9598
	RFE (32/35)	0.9629	0.9633	0.9629	0.9606
	**Lasso (C: 0.1) (25/35)**	**0.9629**	**0.9631**	**0.9629**	**0.9607**
LightGBM	All Features (35/35)	0.9614	0.9616	0.9614	0.9589
	BorutaSHAP (15/35)	0.9593	0.9598	0.9593	0.9563
	**RFE (32/35)**	**0.9614**	**0.9616**	**0.9614**	**0.9589**
	Lasso (C: 0.01) (16/35)	0.9596	0.9600	0.9596	0.9567

**Table 6:** Bayesian optimization comparison for the selected base learners on the WUSTL-EHMS-2020 dataset. Results are reported on the validation subset. For each model, the better of the two compared settings is shown in bold.

Base Learner	Default Hyperparameter Performances				Optimized Hyperparameter Performances			
	Accuracy	Precision	Recall	F1-Score	Accuracy	Precision	Recall	F1-Score
XGBoost	0.9700	0.9697	0.9700	0.9688	**0.9700**	**0.9695**	**0.9700**	**0.9690**
LightGBM	0.9614	0.9616	0.9614	0.9589	**0.9688**	**0.9683**	**0.9688**	**0.9676**
CatBoost	0.9629	0.9631	0.9629	0.9607	**0.9721**	**0.9717**	**0.9721**	**0.9713**

**Table 7:** Meta-learner comparison for the stacking stage on the WUSTL-EHMS-2020 dataset. Results are reported on the validation subset. The selected configuration is shown in bold.

Meta Learner	Accuracy	Precision	Recall	F1-Score
**RandomForest**	**0.9782**	**0.9779**	**0.9782**	**0.9780**
XGBoost	0.9755	0.9752	0.9755	0.9753
LightGBM	0.9761	0.9758	0.9761	0.9759
GradientBoost	0.9749	0.9745	0.9749	0.9746

**Table 8:** Per-class results and confusion matrix of D3P on the WUSTL-EHMS-2020 test set for the run with seed 42. In the confusion matrix, rows correspond to the true class and columns to the predicted class.

					Predicted Class	
Class	Precision	Recall	F1-Score	Support	Benign	Attack
Benign	0.9780	0.9877	0.9828	2842	2807	35
Attack	0.9109	0.8504	0.8796	421	63	358
Macro	0.9445	0.9190	0.9312			
Weighted	0.9694	0.9700	0.9695			

**Table 9:** Train set statistics after balancing for the CICIoMT2024 dataset.

Class	Original Train Set	Balanced Train Set
DDoS	3,823,350	192,524
DoS	1,444,423	192,493
MQTT	210,350	191,723
Benign	154,186	192,564
Recon	79,416	190,178
Spoofing	12,838	190,083

**Table 10:** Comparison of base learner performances on the original and balanced datasets. Results are reported on the validation subset. The balanced dataset was obtained using the SMOTETomek-LVQ-SMOTE strategy. Values that outperform those obtained on the original dataset are shown in bold.

Model	Original				Balanced			
	Acc.	Prec.	Rec.	F1	Acc.	Prec.	Rec.	F1
Decision Tree	**0.9978**	**0.9983**	**0.9978**	**0.9980**	0.9977	0.9978	0.9977	0.9977
CatBoost	0.9510	0.9542	0.9510	0.9494	**0.9982**	**0.9982**	**0.9982**	**0.9982**
Random Forest	0.9981	0.9981	0.9981	0.9981	0.9981	0.9981	0.9981	0.9981
Extra Trees	0.9965	0.9965	0.9965	0.9965	**0.9967**	**0.9967**	**0.9967**	**0.9967**
XGBoost	0.9973	0.9973	0.9973	0.9973	**0.9974**	**0.9975**	**0.9974**	**0.9974**
LightGBM	0.9954	0.9958	0.9954	0.9955	**0.9975**	**0.9976**	**0.9975**	**0.9975**
GradientBoost	0.9960	0.9961	0.9960	0.9961	**0.9975**	**0.9975**	**0.9975**	**0.9975**

**Table 11:** Feature selection comparison for the selected base learners on the CICIoMT2024 dataset. Results are reported on the validation subset. The configuration selected for each base learner is shown in bold.

Base Learner	Feature Selection Method	Accuracy	Precision	Recall	F1-Score
Decision Tree	All Features (45/45)	0.9977	0.9978	0.9977	0.9977
	**RFE (8/45)**	**0.9977**	**0.9977**	**0.9977**	**0.9977**
	BorutaSHAP (24/45)	0.9974	0.9977	0.9974	0.9976
	L1/Lasso (C: 0.1) (44/45)	0.9977	0.9978	0.9977	0.9977
Random Forest	All Features (45/45)	0.9981	0.9981	0.9981	0.9981
	BorutaSHAP (44/45)	0.9980	0.9980	0.9980	0.9980
	RFE (10/45)	0.9976	0.9978	0.9976	0.9977
	**L1/Lasso (C: 0.01) (42/45)**	**0.9982**	**0.9982**	**0.9982**	**0.9982**
CatBoost	All Features (45/45)	0.9982	0.9982	0.9982	0.9982
	**BorutaSHAP (32/45)**	**0.9981**	**0.9982**	**0.9981**	**0.9982**
	RFE (40/45)	0.9977	0.9979	0.9977	0.9978
	L1/Lasso (C: 0.01) (42/45)	0.9981	0.9982	0.9981	0.9982

**Table 12:** Hyperparameter comparison for the selected base learners on the CIC IoMT 2024 dataset. Results are reported on the validation subset. For each model, the better of the two compared settings is shown in bold.

Base Learner	Default Hyperparameter Performances				Optimized Hyperparameter Performances			
	Accuracy	Precision	Recall	F1-Score	Accuracy	Precision	Recall	F1-Score
CatBoost	**0.9981**	**0.9982**	**0.9981**	**0.9982**	0.9953	0.9953	0.9953	0.9953
Decision Tree	0.9977	0.9977	0.9977	0.9977	**0.9981**	**0.9981**	**0.9981**	**0.9981**
Random Forest	0.9982	0.9982	0.9982	0.9982	0.9981	0.9983	0.9981	0.9982

**Table 13:** Meta-learner comparison for the stacking stage on the CIC IoMT 2024 dataset. Results are reported on the validation subset. The selected configuration is shown in bold.

Meta Learner	Accuracy	Precision	Recall	F1-Score
**RandomForest**	**0.9985**	**0.9985**	**0.9985**	**0.9985**
XGBoost	0.9984	0.9985	0.9984	0.9985
LightGBM	0.9984	0.9985	0.9984	0.9984
GradientBoost	0.9984	0.9984	0.9984	0.9984

**Table 14:** Comparison of studies reporting results on the WUSTL-EHMS-2020 datasets. N/R: Not reported for that dataset in the cited source; WUSTL values are taken from the cited study reports.

Study	Method	Setting	Dataset	Accuracy	Precision	Recall	F1-Score
Aljuhani et al. [5]	PSO-DT (SaaS-based Explainable IDS)	Table 2 results	WUSTL-EHMS-2020	96.57%	97.78%	98.26%	98.02%
Hady et al. [42]	RF	Table 11 From [20]	WUSTL-EHMS-2020	92.13%	N/R	N/R	N/R
Hady et al. [42]	KNN	Table 11 From [20]	WUSTL-EHMS-2020	92.00%	N/R	N/R	N/R
Hady et al. [42]	SVM	Table 11 From [20]	WUSTL-EHMS-2020	92.40%	N/R	N/R	N/R
Hady et al. [42]	DNN	Table 11 From [20]	WUSTL-EHMS-2020	90.04%	N/R	N/R	N/R
Dina et al. [43]	FNN	Table 11 From [20]	WUSTL-EHMS-2020	93.08%	93.82%	73.59%	79.75%
Dina et al. [43]	FNN-Focal	Table 11 From [20]	WUSTL-EHMS-2020	93.26%	95.24%	73.69%	80.11%
Dina et al. [43]	CNN	Table 11 From [20]	WUSTL-EHMS-2020	92.86%	92.84%	73.27%	79.27%
Dina et al. [43]	CNN-Focal	Table 11 From [20]	WUSTL-EHMS-2020	93.08%	94.23%	73.38%	79.63%
Ravi et al. [23]	CNN-LSTM (network features)	10-fold CV, Section 2 summary	WUSTL-EHMS-2020	95.00%	94.00%	85.00%	88.00%
Ravi et al. [23]	CNN-LSTM (biometric features)	10-fold CV, Section 2 summary	WUSTL-EHMS-2020	88.00%	75.00%	76.00%	75.00%
Ravi et al. [23]	CNN-LSTM (combined network + biometric features)	10-fold CV, Section 2 summary	WUSTL-EHMS-2020	99.00%	99.00%	98.00%	98.00%
Naeem et al. [25]	Deep Ensemble (Transformer + self-attention DCNN + LSTM)	Table 4 results	WUSTL-EHMS-2020	94.16%	94%	78%	84%
Zukaib et al. [28]	Meta-IDS (signature-based)	Table 7 results	WUSTL-EHMS-2020	99.57%	99.56%	99.57%	99.56%
Zukaib et al. [28]	Meta-IDS (anomaly-based)	Table 9 results	WUSTL-EHMS-2020	99.5%	99.5%	99.5%	99.5%
Zhang et al. [44]	XGB	Table 11 From [20]	WUSTL-EHMS-2020	95.01%	94.94%	95.01%	94.64%
Zhang et al. [44]	RF	Table 11 From [20]	WUSTL-EHMS-2020	93.47%	93.60%	93.47%	92.66%
Zhang et al. [44]	KNN	Table 11 From [20]	WUSTL-EHMS-2020	93.66%	93.33%	93.66%	93.08%
Zhang et al. [44]	SVM	Table 11 From [20]	WUSTL-EHMS-2020	93.08%	93.44%	93.08%	92.02%
Kilincer et al. [51]	MLP	Table 11 From [20]	WUSTL-EHMS-2020	96.20%	96.19%	96.16%	96.17%
Pinar et al. [20]	KNN	Table 11 From [20]	WUSTL-EHMS-2020	94.45%	90.59%	82.59%	86.41%
Pinar et al. [20]	SVM	Table 11 From [20]	WUSTL-EHMS-2020	93.95%	96.73%	75.90%	85.06%
Pinar et al. [20]	XGB	Table 11 From [20]	WUSTL-EHMS-2020	94.64%	96.80%	78.78%	86.87%
Pinar et al. [20]	DNN	Table 11 From [20]	WUSTL-EHMS-2020	96.37%	95.18%	87.71%	91.30%
Kumari et al. [45]	Variational Autoencoder (VAE)	Table 1 results	WUSTL-EHMS-2020	91.61%	91.69%	91.61%	91.60%
Liu [46]	BiGRU/RBWK	Table 12 results	WUSTL-EHMS-2020	95.60%	96.2%	96.5%	96.3%
Nasayreh et al. [47]	LSTM + PCA + KNN (proposed scheme)	Table 8 results	WUSTL-EHMS-2020	99.90%	99.90%	99.90%	99.90%
Sharma and Shambharkar [30]	MA-DeepCRNN (Multi-attention DeepCRNN)	Table 17 average cross-validation	WUSTL-EHMS-2020	94.11%	92.40%	91.82%	92.11%
Proposed Method	D3P (RandomForest meta-learner; XGBoost + LightGBM + CatBoost), all 35 input features	Test set, mean ± std over five seeds	WUSTL-EHMS-2020	97.01 ± 0.15%	96.94 ± 0.19%	97.01 ± 0.15%	96.97 ± 0.14%
Proposed Method	D3P without Packet_num (34 input features)	Test set, mean ± std over five seeds	WUSTL-EHMS-2020	94.91 ± 0.17%	94.78 ± 0.21%	94.91 ± 0.17%	94.56 ± 0.17%

**Table 15:** Comparison of studies reporting results on the CICIoMT2024 datasets. Values marked with an asterisk are weighted metrics reported in the cited studies. N/R: Not reported in the cited study table.

Study	Method/Sub-Method	Setting	Dataset	Class Setting	Accuracy	Precision	Recall	F1-Score
Naeem et al. [25]	Ensemble learning with random forest	Meta Learner Result (Table 4)	CICIoMT2024	16-class	85.86% *	68% *	52% *	55% *
Hossain et al. [32]	Naive Bayes	Reduced feature set	CICIoMT2024	6-class	69.46%	59.70%	69.42%	58.38%
Hossain et al. [32]	Naive Bayes	Full feature set	CICIoMT2024	6-class	67.22%	55.21%	66.83%	57.71%
Hossain et al. [32]	Random Forest	Reduced feature set	CICIoMT2024	6-class	97.62%	96.24%	97.23%	96.83%
Hossain et al. [32]	Random Forest	Full feature set	CICIoMT2024	6-class	96.73%	95.46%	95.22%	95.78%
Hossain et al. [32]	SVM	Reduced feature set	CICIoMT2024	6-class	83.15%	82.57%	83.11%	82.76%
Hossain et al. [32]	SVM	Full feature set	CICIoMT2024	6-class	80.40%	79.27%	80.85%	80.44%
Hossain et al. [32]	MultiD-CNN	Reduced feature set	CICIoMT2024	6-class	98.11%	97.73%	98.21%	97.88%
Hossain et al. [32]	MultiD-CNN	Full feature set	CICIoMT2024	6-class	96.72%	95.56%	96.65%	95.42%
Akar et al. [27]	Logistic Regression	Table 5 result	CICIoMT2024	2-class	98.00%	98.00%	98.00%	98.00%
Akar et al. [27]	DL ANN + Adam	50 epochs	CICIoMT2024	2-class	100.00%	100.00%	100.00%	100.00%
Akar et al. [27]	DL LSTM	50 epochs	CICIoMT2024	2-class	100.00%	100.00%	100.00%	100.00%
Akar et al. [27]	Logistic Regression	Table 6 result	CICIoMT2024	6-class	57.00%	37.00%	57.00%	43.00%
Akar et al. [27]	DL ANN + Adam	50 epochs	CICIoMT2024	6-class	73.00%	73.00%	73.00%	70.00%
Akar et al. [27]	DL LSTM	50 epochs	CICIoMT2024	6-class	98.00%	98.00%	98.00%	98.00%
Alabbadi and Bajaber [31]	X-FuseDNN	Table 4 result	CICIoMT2024	6-class	97.60%	97.59%	97.60%	97.59%
Alabbadi and Bajaber [31]	X-FuseRLSTM	Table 4 result	CICIoMT2024	6-class	98.05%	98.02%	98.05%	98.02%
Hnamte et al. [34]	LWIDS-DCNN	Table 4 result	CICIoMT2024	n/a	99.70%	99.70%	99.73%	99.68%
Shaikh et al. [33]	HCLR-IDS	Table 4 (binary, weighted metrics)	CICIoMT2024	2-class	99.58% *	99.53% *	99.83% *	99.57% *
Shaikh et al. [33]	HCLR-IDS	Table 5 (weighted)	CICIoMT2024	18-class	77.73% *	76.02% *	77.73% *	72.47% *
Hernandez-Jaimes et al. [35]	Random Forest (Nilsimsa Fingerprinting)	From ref. [41]	CICIoMT2024	2-class	99.44%	N/R	N/R	N/R
Dadkhah et al. [48]	Random Forest	From ref. [41]	CICIoMT2024	19-class	73.00%	N/R	N/R	N/R
Naeem et al. [25]	Deep Ensemble (Transformer + DCNN + LSTM)	From ref. [41]	CICIoMT2024	6-class	99.00%	N/R	N/R	N/R
Akar et al. [27]	LSTM (2×LSTM + Dense)	From ref. [41]	CICIoMT2024	19-class	98.00%	N/R	N/R	N/R
Ali et al. (2025) [52]	Vision Transformer (ViT)	From ref. [41]	CICIoMT2024	2-class	99.50%	N/R	N/R	N/R
Mohammadi et al. (2024) [53]	CNN	From ref. [41]	CICIoMT2024	2/6/19	99.00%	N/R	N/R	N/R
Hossain et al. [32]	MultiD-CNN (with IG feature selection)	From ref. [41]	CICIoMT2024	6-class	98.11%	N/R	N/R	N/R
Jeremiah et al. [41]	TabNet + Shallow MLP (Ensemble Multiview FL)	Table 6 result	CICIoMT2024	2/6/18	99.70%/99.40%	N/R	N/R	N/R
Shebl et al. [40]	DCNN	Table 10 result	CICIoMT2024	2-class	99.98%	N/R	N/R	N/R
Shebl et al. [40]	DCNN	Table 10 result	CICIoMT2024	multiclass	99.86%	N/R	N/R	N/R
Alturki and Alsulami [39]	Semi-Supervised Tree-Based + Entropy Filtering (RF)	Table 1 summary	CICIoMT2024	multiclass	98.00%	N/R	N/R	N/R
Alturki and Alsulami [39]	Semi-Supervised Tree-Based + Entropy Filtering (XGB)	Table 1 summary	CICIoMT2024	multiclass	98.00%	N/R	N/R	N/R
Akhi et al. [38]	Res-TCN	Table 11 result	CICIoMT2024	multiclass	98.82%	99.00%	99.00%	99.00%
Akhi et al. [38]	Res-TCN	Table 11 result	CICIoMT2024	2-class	99.90%	100.00%	100.00%	100.00%
Choubey et al. [37]	Deep Isolation Forest (DIF)	Table I result	CICIoMT2024	2-class	N/R	100%	98.1%	99.00%
Gueriani et al. [36]	DNN+(GRU-MHA)	Table IV result	CICIoMT2024	multiclass	98.22%	98.23%	98.22%	98.22%
Proposed Method	D3P (RF Meta Learner, DT + RF + CatBoost)	Test set, mean ± std over five seeds	CICIoMT2024	6-class	99.78 ± 0.04%	99.77 ± 0.05%	99.78 ± 0.05%	99.77 ± 0.05%

## Data Availability

The data presented in this study are available in the WUSTL-EHMS-2020 and CICIoMT2024 public datasets. These data were derived from the following resources available in the public domain: https://www.cse.wustl.edu/~jain/ehms/index.html (accessed on 20 October 2026) and https://www.unb.ca/cic/datasets/iomt-dataset-2024.html (accessed on 20 October 2026).

## Abbreviations

The following abbreviations are used in this manuscript:

ADASYN	Adaptive Synthetic Sampling
BorutaSHAP	Boruta Feature Selection with SHAP Values
CatBoost	Categorical Boosting
CCR	Combined Cleaning and Resampling
D3P	Dynamic Data-Driven Pipeline
DDoS	Distributed Denial of Service
DL	Deep Learning
DoS	Denial of Service
DT	Decision Tree
ENN	Edited Nearest Neighbors
IAT	Inter-Arrival Time
IDS	Intrusion Detection System
IHT	Instance Hardness Threshold
IoMT	Internet of Medical Things
LightGBM	Light Gradient Boosting Machine
LIME	Local Interpretable Model-agnostic Explanations
LVQ	Learning Vector Quantization
ML	Machine Learning
MQTT	Message Queuing Telemetry Transport
NCR	Neighborhood Cleaning Rule
OSS	One-Sided Selection
RF	Random Forest
RFE	Recursive Feature Elimination
SHAP	SHapley Additive exPlanations
SMOTE	Synthetic Minority Over-sampling Technique
XAI	Explainable Artificial Intelligence
XGBoost	eXtreme Gradient Boosting

## Appendix A. Supplementary Tables

This appendix lists the feature subsets selected for each base learner and the final hyperparameter settings on both datasets. Table A1 and Table A2 refer to WUSTL-EHMS-2020, and Table A3 and Table A4 refer to CICIoMT2024.

**Table A1:** Selected features by model-specific best configuration on WUSTL-EHMS-2020 (common features across all three models are shown in bold).

XGBoost (All Features)	CatBoost (Lasso, C: 0.1)	LightGBM (RFE)
SrcBytes	DstBytes	SrcBytes
DstBytes	**SrcLoad**	**SrcLoad**
**SrcLoad**	**SrcGap**	DstLoad
DstLoad	**DstGap**	**SrcGap**
**SrcGap**	**DIntPkt**	**DstGap**
**DstGap**	**DIntPktAct**	SIntPkt
SIntPkt	**SrcJitter**	**DIntPkt**
**DIntPkt**	**sMaxPktSz**	**DIntPktAct**
SIntPktAct	dMaxPktSz	**SrcJitter**
**DIntPktAct**	**dMinPktSz**	DstJitter
**SrcJitter**	**Dur**	**sMaxPktSz**
DstJitter	**Trans**	sMinPktSz
**sMaxPktSz**	**TotPkts**	**dMinPktSz**
dMaxPktSz	**Loss**	**Dur**
sMinPktSz	**pSrcLoss**	**Trans**
**dMinPktSz**	**pDstLoss**	**TotPkts**
**Dur**	**Packet_num**	TotBytes
**Trans**	**Temp**	Load
**TotPkts**	**SpO2**	**Loss**
TotBytes	**Pulse_Rate**	pLoss
Load	**SYS**	**pSrcLoss**
**Loss**	**DIA**	**pDstLoss**
pLoss	**Heart_rate**	Rate
**pSrcLoss**	**Resp_Rate**	**Packet_num**
**pDstLoss**	**ST**	**Temp**
Rate		**SpO2**
**Packet_num**		**Pulse_Rate**
**Temp**		**SYS**
**SpO2**		**DIA**
**Pulse_Rate**		**Heart_rate**
**SYS**		**Resp_Rate**
**DIA**		**ST**
**Heart_rate**		
**Resp_Rate**		
**ST**		

**Table A2:** Best optimized hyperparameters for the selected base learners on WUSTL-EHMS-2020.

Model	Hyperparameter	Best Value
CatBoost	iterations	295
	depth	6
	learning_rate	0.3
	l2_leaf_reg	0.6252
	border_count	254
XGBoost	n_estimators	477
	max_depth	7
	learning_rate	0.3
	subsample	1.0
	colsample_bytree	1.0
	min_child_weight	1
	reg_lambda	6.4447
LightGBM	n_estimators	282
	learning_rate	0.2208
	num_leaves	16
	min_child_samples	14
	subsample	0.5144
	colsample_bytree	0.9415

**Table A3:** Selected features by model-specific best configuration on CICIoMT2024 (common features across all three models are shown in bold).

Decision Tree (RFE, 8/45)	Random Forest (Lasso, C: 0.01, 42/45)	CatBoost (BorutaSHAP, 32/45)
**Rate**	Header_Length	psh_flag_number
**syn_flag_number**	Protocol Type	HTTPS
**ack_flag_number**	Duration	Srate
**ack_count**	**Rate**	Magnitue
**rst_count**	Srate	Weight
SSH	fin_flag_number	Std
**Max**	**syn_flag_number**	Min
**IAT**	rst_flag_number	fin_count
	psh_flag_number	TCP
	**ack_flag_number**	AVG
	cwr_flag_number	**rst_count**
	**ack_count**	ICMP
	syn_count	Protocol Type
	fin_count	SSH
	**rst_count**	syn_count
	HTTP	**Rate**
	HTTPS	DHCP
	DNS	Radius
	Telnet	UDP
	SMTP	Duration
	IRC	**ack_flag_number**
	TCP	Variance
	UDP	fin_flag_number
	DHCP	**ack_count**
	ARP	Tot sum
	ICMP	Number
	IGMP	**syn_flag_number**
	IPv	DNS
	LLC	**IAT**
	Tot sum	Header_Length
	Min	Tot size
	**Max**	**Max**
	AVG	
	Std	
	Tot size	
	**IAT**	
	Number	
	Magnitue	
	Radius	
	Covariance	
	Variance	
	Weight	

**Table A4:** Final hyperparameter settings for the selected base learners on CIC IoMT 2024. Random Forest and CatBoost are reported at their default settings.

Model	Hyperparameter	Value
Decision Tree	max_depth	21
	min_samples_split	8
	min_samples_leaf	1
	max_features	1.0
Random Forest	estimators	(Default)
	max_depth	(Default)
	min_samples_split	(Default)
	min_samples_leaf	(Default)
	max_features	(Default)
CatBoost	iterations	(Default)
	depth	(Default)
	learning_rate	(Default)
	l2_leaf_reg	(Default)
	border_count	(Default)

## Appendix B. Supplementary Explainability Analyses

Figure A1 complements the global SHAP ranking in Figure 3 by showing the signed SHAP-value distribution of each feature for the three optimized base learners on WUSTL-EHMS-2020, which reveals not only how important a variable is but also how it contributes across samples. Figure A2 shows the corresponding SHAP interaction-value matrices. In all three learners, the largest values lie on the diagonal, so main effects dominate, but off-diagonal entries show that timing and volume variables such as DIntPkt, SrcJitter, and Packet_num also interact with other features. The interaction structure differs between the learners. LightGBM concentrates on a few high-intensity pairs, CatBoost spreads its interactions over many feature pairs, and XGBoost lies between the two, which illustrates that the learners encode feature relationships differently.

Figure A3 presents SHAP beeswarm plots for the Spoofing class on CICIoMT2024. Points on the positive side push the prediction toward Spoofing and points on the negative side push it away, while the color indicates the feature value. In all three models, IAT, Header_Length, and count-related features such as Number and fin_count show a wide spread, so temporal variation and traffic intensity are the main predictors of Spoofing. CatBoost shows the largest SHAP range with asymmetric tails, Random Forest distributes its effect more evenly across header and flag features, and Decision Tree relies on a few splitting features such as IAT and Max. The learners therefore encode Spoofing-related information in different ways that the stacking stage can combine.
